# Structural basis for transport and inhibition of nucleotide sugar transport in pathogenic fungi

**DOI:** 10.1038/s41467-026-72729-6

**Published:** 2026-05-02

**Authors:** Joanne L. Parker, Justin C. Deme, Bjarne Feddersen, Susan M. Lea, Simon Newstead

**Affiliations:** 1 Department of Biochemistry, University of Oxford, Oxford, UK; 2 The Kavli Institute for Nanoscience Discovery, University of Oxford, Oxford, UK; 3 Center for Structural Biology, Center for Cancer Research, National Cancer Institute, Frederick, MD USA; 4 Department of Structural Biology, St. Jude Chrildren’s Research Hospital, 262 Danny Thomas Place, Memphis, TN USA

**Keywords:** Cryoelectron microscopy, Glycobiology, Membrane structure and assembly

## Abstract

GDP-Mannose transporters are Golgi-localised solute carriers that are essential for the virulence of pathogenic fungi, serving as critical components of fungal glycosylation pathways. However, the mechanism by which nucleotide sugars are recognised and transported across the Golgi membrane remains unclear, hindering efforts to develop effective inhibitors that could serve as distinct antifungal agents. Here, we present cryo-EM structures of the GDP-Mannose transporter, Vrg4, from *Candida albicans* in complex with nanobodies in both the cytoplasmic and Golgi-facing states. Structural comparisons between these two states, in addition to a GDP-mannose bound structure, demonstrate the importance of ligand movement during transport. Additionally, we demonstrate the ability of the nanobodies to specifically inhibit Vrg4, presenting proof-of-principle that nanobodies can be used as effective inhibitors of nucleotide sugar transport and glycosylation in cells.

## Abstract (web summary)

Invasive fungal pathogens have become a significant concern in healthcare systems worldwide. Here, authors demonstrate that nanobodies targeting the GDPmannose transporter can arrest fungal cell growth. The structures also reveal the complete transport cycle for an SLC35 nucleotide sugar transporter.

## Introduction

Invasive fungal pathogens have become a significant concern in healthcare systems worldwide. Fungal skin infections affect one in three people and cause over a million deaths each year. Candidiasis, caused by the overgrowth of mainly *Candida albicans*, accounts for approximately 70% of fungal infections^1,2^. A particular concern is the rising mortality rate among immunocompromised patients with Candidiasis, which is now nearly 40%^1^. For invasive fungal infections, only four major classes of antifungal agents exist^3^. However, poor bioavailability, host toxicities, and the widespread emergence of resistance are driving efforts to identify distinct targets and methods for inhibiting fungal growth^4,5^.

The cell wall of fungi mainly consists of glycomannosylated conjugates that create a protective layer against the human immune system^6^. GDP-mannose is a nucleotide sugar metabolite that serves as the substrate for glycosyltransferase enzymes that build the fungal cell wall^7,8^. GDP-mannose transporters are essential for cell survival in fungal species^9–12^. In addition, the transport of GDP-mannose into the Golgi is crucial for virulence in pathogenic fungi, as well as in parasitic protozoa such as the Trypanosomatidae family^13,14^. However, no antifungal or antiprotozoal drugs currently target GDP-mannose transport, despite these pathways offering promising new directions for drug development.

Nucleotide sugar transporters (NSTs) belong to the Drug and Metabolite Transporter (DMT) superfamily^15^, which in humans forms part of the SLC35 family of solute carriers^16^. Nucleotide sugar transporters operate via a strict antiport mechanism, moving nucleotide sugars into the Golgi in exchange for the free nucleotide monophosphate, which is released back into the cytoplasm^17^. To date, only six structures of DMT family transporters have been reported, recognising a range of metabolites and drugs, including the *Plasmodium falciparum* chloroquine transporter^18^, the bacterial amino acid transporter YddG^19^, and the human ATP/ADP exchanger^20^. However, only three representative examples of NSTs have been described: the murine CMP-sialic acid transporter^21^, a plant nucleotide transporter that recognises CMP-sialic acid^22^, and Vrg4, the GDP-mannose transporter from *Saccharomyces cerevisiae*^23,24^. These structures reveal that the DMT family shares a conserved 10-transmembrane (TM) architecture, arranged as two inverted topology repeats of five TM helices^25^. Nevertheless, all three NST structures were captured in the Golgi-facing state, which limits insights into how these transporters recognise ligands from the cytoplasm and undergo alternating access transport.

Here, we present the cryo-EM structures of the GDP-mannose transporter, Vrg4, from the pathogenic fungus *Candida albicans*, in complex with nanobodies, in both the Golgi- and cytoplasm-facing conformations. Nanobodies, also known as single-chain antibodies, are increasingly being investigated as potential therapeutic agents across a broad range of medical fields, including cancer, inflammatory conditions, infectious diseases, and neurological disorders^26^. Our functional assays provide proof-of-concept data showing that nanobodies can be expressed within yeast cells and used to selectively inhibit the *C. albicans* GDP-mannose transporter CaVrg4. Finally, structures of CaVrg4 in the cytoplasmic-facing state bound to GDP-mannose provide important mechanistic insight into the alternating access transport mechanism within NSTs, with broader implications for understanding transport within the wider SLC35 family^16^.

## Results

### Identification of inhibitory nanobodies specific for the GDP-mannose transporter from *C. albicans*

**Fig. 1:**
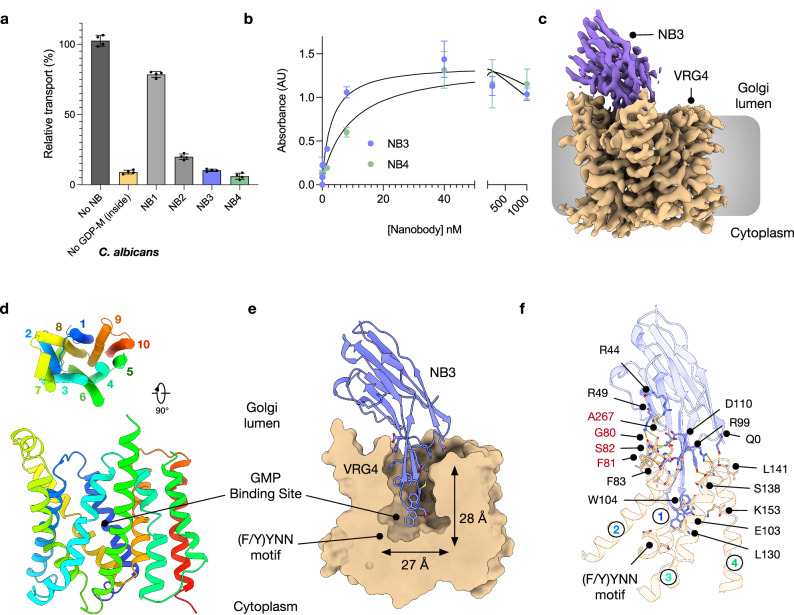
Analysis of inhibitory nanobodies to C. albicans Vrg4. **a** Transport assay data showing the uptake of GMP via *C. albicans* Vrg4 (CaVrg4) in the presence of nanobodies (NB) relative to a no NB control in liposomes. Without GDP-mannose on the inside of the liposome (no GDP-M inside), this transporter cannot function. (*n* = 4 independent experiments performed on different days, the mean is shown, and errors indicate SD). **b** ELISA data showing normalised absorbance (normalised to the absorbance at 1 μM for each NB) against NB concentration. The calculated K_D_ for NB3 is ~3 nM and for NB4 is ~10 nM. (*n* = 3 independent experiments performed on different days, the mean is shown, and errors indicate SD). Source data (for a and b) are provided as a Source Data file **c** Cryo-EM density of the CaVrg4 NB3 complex (contoured at a threshold of 0.476). The Golgi membrane is represented as a grey box. **d** Cartoon representation of CaVrg4 in the Golgi facing conformation, with helices coloured from blue to red from the N-terminus. **e** Surface representation of the cryo-EM structure of CaVrg4 in complex with NB3. The CDR3 loop of NB3 extends into the substrate-binding site of the transporter. Shown as sticks are the interacting side chains and the two rotamer positions observed for Trp104. The (F/Y)YNN^247^ motif for nucleotide specificity and the GMP-binding site are labelled. Distances shown represent the size of the substrate binding cavity. **f** Zoomed in view of the interactions observed between NB3 (blue) and Vrg4 (wheat). Side chains unique to CaVrg4 are shown in red. View is equivalent to panel (**e**).

To identify distinct ways to inhibit the GDP-mannose transporter, Vrg4, we immunised llamas with the Vrg4 protein from the pathogenic yeast *Candida albicans*. A library of nanobodies generated from the immunisation was obtained, and a subset was screened for the ability to inhibit the activity of CaVrg4 in vitro (Supplementary information Fig. 1a, b). We identified four nanobodies that exhibited the ability to block the uptake of ^3^H GMP into liposomes containing CaVrg4, with two nanobodies, NB3 and NB4, showing full inhibition of transport function (Fig. 1a). Nanobodies NB3 and NB4 displayed the highest affinity (K_D_) towards purified CaVrg4, of ~ 3 nM and 10 nM, respectively (Fig. 1b, Supplementary information Fig. 1c, d). However, neither NB3 nor NB4 showed any detectable inhibition against Vrg4 from *S. cerevisiae* nor *Candida auris*, which is 84% similar and 77% identical to the *C. albicans* homologue (Supplementary information Fig. 1e, f), highlighting the specific nature of these nanobodies towards *C. albicans*.

### Structural basis for inhibition of *C. albicans* Vrg4

To understand the mode of inhibition, we next determined the structure of CaVrg4 with both NB3 and NB4. Vrg4 is only 37 kDa and, therefore, a relatively small membrane protein for structure determination using cryo-EM. Nevertheless, we determined the structure of Vrg4 in complex with NB3 at 3.0 Å (Fig. 1c, d, Table 1 and Supplementary information Figs. 2, 3). The structure adopts a similar Golgi-facing state as previously observed in the crystal structure of *S. cerevisiae* Vrg4 (PDB:5OGE)^24^ with an r.m.s.d of 1.47 Å over 288 C_α_ atoms. The CDR3 loop of NB3 forms an extended beta-hairpin, which extends 23 Å into the GDP-mannose binding cavity (Fig. 1e). The loop region of the CDR3 hairpin interacts with regions within the binding site that are important for GDP-mannose and GMP recognition^24,27^. Specifically, Glu103 in the CDR3 loop forms a salt bridge interaction with Lys153 on TM4 and sits close to Tyr311 on TM9 (Fig. 1f, Supplementary information Fig. 4a) and is essential for *S. cerevisiae* Vrg4 function^23,24^. Both TM4 and TM9 play important roles in switching the transporter between cytoplasmic and Golgi lumen-facing states during transport, as we discuss below. Sitting next to Glu103 on the CDR3 loop is Trp104, which is located within ~ 5 Å of the conserved (F/Y)YNN^247^ motif on TM7, which is responsible for guanine recognition. Interestingly, Trp104 adopts two rotamer positions, with one rotamer engaging the guanine pocket and the other packing against Leu130 and Ile134 on TM3, which also forms another of the alternating access gating helices. In total, the CDR3 loop makes six direct interactions to key regions of the GDP-mannose binding site with one additional interaction mediated by a water molecule (Supplementary information Fig. 4a, Supplementary information Table 1). However, all of these interactions are to side chains that are conserved within the binding site of closely related fungal GDP-mannose transporters, such as Vrg4 from *C. auris* and *S. cerevisiae* (Supplementary information Table 1).

**Table 1:** Cryo-EM data collection, refinement, and validation statistics

	CaVrg4-NB3 (EMDB-54523) (PDB 9S35)	CaVrg4-NB4- GDP-mannose (EMDB-54524) (PDB 9S36)
**Data collection and processing**		
Magnification	165,000	165,000
Voltage (kV)	300	300
Electron exposure (e–/Å^2^)	55.0	53.7
Defocus range (μm)	−2.0 to −0.5	−2.0 to −0.8
Pixel size (Å)	0.732	0.732
Symmetry imposed	C1	C1
Initial particle images (no.)	7,870,980	8,921,772
Final particle images (no.)	348,612	51,142
Map resolution (Å) FSC threshold	3.0 0.143	3.4 0.143
Map sharpening (Å^2^)	105.9	80.2
**Refinement**		
Initial model used (PDB code)	ScVrg4 (5OGK)	
Model composition in the asymmetric unit Non-hydrogen atoms Protein residues Ligands	3361 428 0	3455 441 1
Average *B* factors (Å^2^) Protein Ligand	26.53	52.70 95.50
R.m.s. deviations Bond lengths (Å) Bond angles (°)	0.004 0.534	0.002 0.463
Ramachandran % (favoured) (outliers)	97.5 0	96.57 0
Validation MolProbity score Clashscore Poor rotamers (%)	1.63 4.88 2.19	1.39 3.75 0.81

The high number of interactions between NB3 and the conserved NST binding site raised the question of how specificity is achieved for the *C. albicans* transporter. However, NB3 makes several additional interactions with backbone carbonyl groups in the loop between TMs 1 and 2 (Fig. 1f); this region of the transporter shows a high level of sequence diversity across Vrg4 sequences (Supplementary information Fig. 1f, Supplementary information Fig. 4a) and hence, while the CDR3 hairpin interacts with highly conserved regions of the transporters, it is the CDR1 and CDR2 regions that drive specificity in this nanobody. It is interesting that specificity is driven through interactions between the nanobody and the backbone carbonyl groups in the TM1-2 loop region rather than through the side chains. As the backbone of the transporter in this region is noticeably different between the *C. albicans* and *S. cerevisiae* Vrg4 transporters, this finding reveals the ability of nanobodies to recognise subtle differences in structure between otherwise closely related transporters.

**Fig. 2:**
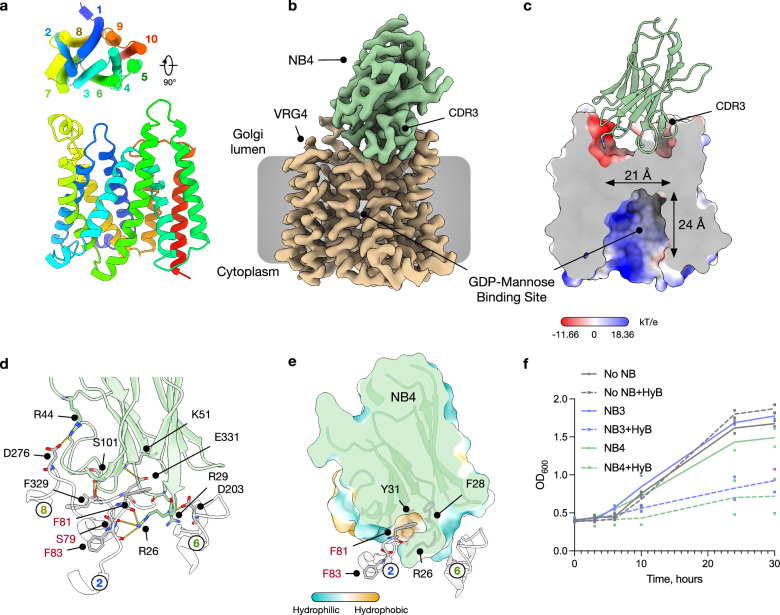
Cytoplasmic facing conformation of Vrg4 with NB4. **a** Cartoon representation of CaVrg4 in the cytosolic facing state, with helices coloured from blue to red from the N-terminus. **b** Cryo-EM density of the CaVrg4 NB4 complex contoured (threshold level of 0.476). The Golgi membrane is represented as a grey box, and the CDR3 loop on NB4 and the GMP-mannose binding site in CaVrg4 are labelled. **c** Electrostatic surface representation of the cryo-EM structure of CaVrg4 in complex with NB4. Distances shown indicate the size of the substrate binding cavity. **d** Zoomed in view of the interacting region of NB4 with CaVrg4. Shown as sticks are the side chains that contribute to the electrostatic surface. Also shown is the position of Phe81, which sits into a pocket on NB4. **e** Zoomed in view of the hydrophobic pocket formed within NB4 that accommodates Phe81 from the TM1-TM2 loop in CaVrg4. NB4 is shown in surface representation and coloured according to hydrophobicity. Side chains unique to CaVrg4 are coloured red. **f** Growth curves of yeast strain NDY5 overexpressing *C. albicans* Vrg4 and either NB3, NB4 or no NB in the presence (+) and absence of hygromycin B (HyB). In the presence of both NB3 and NB4, the sensitivity of this strain to HyB manifests, indicating the inhibition of the function of *C. albicans* Vrg4. (*n* = 2 independent experiments performed on different days, the mean is shown, and errors indicate SD). Source data are provided as a Source Data file.

Obtaining the structure of CaVrg4 in complex with NB4, however, proved more challenging due to a high proportion of uncomplexed particles following the addition of the two proteins prior to grid preparation. However, following enrichment of the sample for the presence of NB4 (see “methods”), we were able to determine the structure to 3.4 Å (Fig. 2a, b, Table 1, Supplementary information Fig. 4b). Although NB4 binds on the same luminal side of the transporter as NB3, it stabilises the opposing cytoplasmic-facing state (Fig. 2c). However, NB4 makes only six hydrogen bond interactions to the transporter (Fig. 2d, Supplementary information Table 2). Most notable is the interaction between Arg26 in CDR1 with the exposed carbonyl groups from Ser79, Phe81 and Phe83 in the same TM1-TM2 loop region observed in the NB3 binding pose, discussed above. Interestingly, the movement of TMs 1 and 2 to adopt the luminally closed state of the transporter results in the repositioning of the TM1-TM2 loop such that the side chain of Phe81 now projects into a hydrophobic pocket formed between the CDR1 and CDR3 regions in NB4. This cavity is absent in NB3 and is created by the repositioning of CDR3 to one side of the nanobody scaffold (Fig. 2b, c, Supplementary information Fig. 5b). In this position, the side chain of Phe81 is completely buried into the pocket formed within the NB4 scaffold (Fig. 2e). The pocket is lined by Phe28 and Tyr31, both of which are unique to the CDR1 region of NB4, with Arg26 sitting beneath Phe81 and completing the clamp around the TM1-2 loop region. In addition to the hydrogen bond and hydrophobic interactions, NB4 makes three salt bridge interactions to the transporter. Arginine 29, which is also part of the CDR1, interacts with Asp203 on TM6, while Lys51 interacts with Glu331 on the TM9-TM10 loop. The third salt bridge is formed by Arg44 and Asp276 on TM8.

The remainder of the CDR3 loop is composed primarily of hydrophobic side chains, which, as mentioned above, loop away from the transporter to pack against the nanobody scaffold (Supplementary information Fig. 5b). This unusual positioning of the CDR3 loop creates a stable platform for the CDR3 region to bind between a cavity located between TM 8 and TM9 on the outside of the transporter. This cavity is created by the movement of TM 1 towards TM3, discussed below, which is a feature of the cytoplasmic open state. Within the CDR3 loop, only Ser101 makes a direct hydrogen bond interaction with the transporter, via the carbonyl groups on Ile328 and Phe329 on TM9 (Fig. 2d, Supplementary information Table 2). Within all the interactions discussed above, only Ser79, Phe81 and Phe83 are unique to CaVrg4, indicating that the main driver for specificity within NB4 is likely to originate from the unique TM1-TM2 loop region that differs in both sequence and structure between the fungal NSTs and the creation of the binding pocket between TM8 and TM9 in the cytoplasmic open state.

Having established the mechanism through which NB3 and NB4 interact with *Ca*Vrg4, we next wanted to test whether NB3 and NB4 could inhibit the transporter in vivo. For this experiment, we used a strain of *S. cerevisiae* containing a mutant version of the *VRG4* gene (Ala286Asp in the GALNK motif), which confers sensitivity to the antifungal hygromycin B in the yeast^28^. Hygromycin B sensitivity can be alleviated through the presence of a functional copy of Vrg4 and is complemented by the *C. albicans VRG4* gene^28^ (Supplementary information Fig. 6a, b). In this complemented yeast strain, we transformed a plasmid containing either NB3 or NB4 with a 5’ secretion signal sequence to target it to the lumen of the Golgi, where our structures indicated both nanobodies interact with CaVrg4. The yeast was grown in minimal media to select for the plasmids, and the expression of the nanobody was induced with galactose. After overnight galactose induction, the yeast culture was diluted, and growth was monitored over 30 h. The results indicate that in the presence of either NB3 or NB4, hygromycin B causes a slow growth phenotype (Fig. 2f, Supplementary information Fig. 6). Our growth assays therefore demonstrate that both NB3 and NB4 suppress yeast growth in the presence of hygromycin B and are thus capable of inhibiting CaVrg4 in vivo.

### Structural comparison between the Golgi and cytosol-facing states

**Fig. 3:**
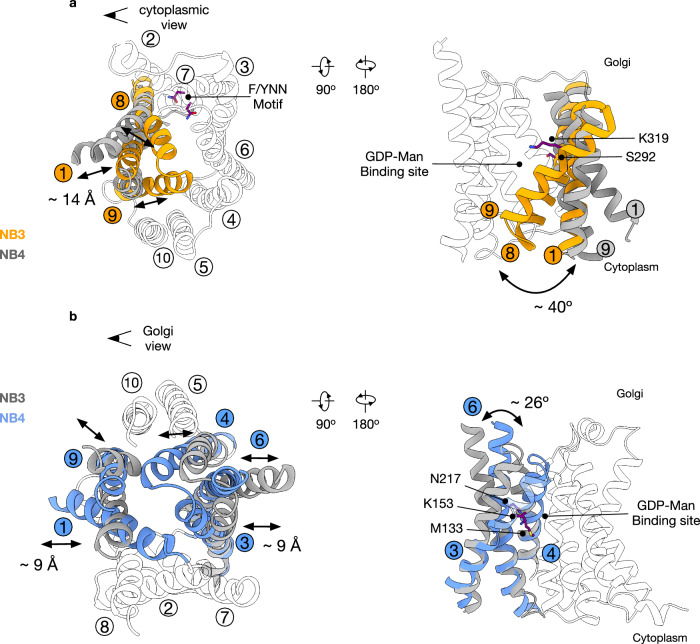
Conformational changes between Golgi and cytoplasmic facing states. **a** Overlay of Vrg4 in the Golgi open (NB3 bound, orange) and cytoplasmic open (NB4 bound, grey) states, highlighting the movement of TMs 1, 8 and 9 (as viewed from the cytoplasm). Residues important for function are indicated as colour sticks and labelled. **b** Overlay of Vrg4 in the cytoplasm open (NB4 bound, blue) and Golgi open (NB3 bound, grey) states, highlighting the movement of TM’s 1, 3, 4, 6 and 9 (as viewed from the Golgi side). Residues important for nucleotide-sugar recognition are indicated as coloured sticks and labelled.

NSTs consist of 10 TM helices that are arranged as two inverted topology repeats of five TMs, such that TMs 1-5 can be superimposed on TMs 6–10 via a 180° rotation in the plane of the membrane^19,24^. Previously, we postulated that transport occurs through the conformational switching of TMs 1, 3 and 4 with TMs 6, 8 and 9, which resulted in the symmetrical pivoting around the central ligand binding site. These structural changes are coordinated with ligand binding through conserved lysines on TMs 4 and 9, which interact with the beta-phosphate and mannose sugar^24^. However, this mechanism was based only on the outward open conformation of *S. cerevisiae* Vrg4 (Golgi lumen-facing) and a symmetrical repeat swapped model of the cytoplasmic-facing state^24^. Our additional structures now allow for the direct comparison of the structural rearrangements required for nucleotide-sugar/nucleotide monophosphate exchange. When viewed from the cytoplasmic side of the Golgi membrane we observe that rather than a symmetrical movement of the gating helices, the predominant movement occurs in TMs 1, 8 and 9, with the remaining helices remaining largely static (Fig. 3a). Specifically, to close the cytoplasmic side of the transporter, TMs 1, 8 and 9 move inwards by ~14 Å and rotate ~ 40° towards TMs 4, 6 and 7. It is important to note that TM7 contains the conserved nucleotide-binding FYNN^247^ motif, which plays an important role in recognising the nucleotide group of the ligand^24^. Thus, the transporter effectively holds the nucleotide in one position whilst the gating helices alternately move to expose the ligand to either the Golgi or cytoplasmic side of the membrane, as discussed in more detail below. Two of the gating helices contain Ser292 (TM8) and Lys319 (TM9), which were previously identified to interact with the ribose group and sugar moiety, respectively^24^. Mutation of Ser292 to alanine reduces transport (Supplementary information Fig. 7a), and the equivalent serine on TM8 in *S. cerevisiae* Vrg4 (Ser266) is important for GMP recognition. The lysine on TM9 forms part of the conserved GALNK^319^ motif, which is important for sugar recognition within the NST superfamily^24^. Taken together, the structural rearrangements of TMs 1, 8 and 9 on the cytoplasmic side of the transporter suggest a mechanism whereby the GDP-mannose is recognised and held in place by the largely static (F/Y)YNN^247^ motif on TM7, whilst the engagement of the ribose and sugar group with TMs 8 and 9 facilitate the structural rearrangements that close the cytoplasmic gate towards the occluded state and luminal release.

In comparison, on the Golgi-facing side of the membrane, the conformational changes are more extensive but smaller (Fig. 3b). To open the Golgi side of the transporter, TMs 1 and 9 move outward by approximately 9 Å, while TM8 remains static. Facing TMs 1 and 9, TMs 3, 4, and 6 also move outward by about 9 Å and rotate away by approximately 26°, pivoting around Met133 (TM3), Lys153 (TM4), and Asn217 (TM6) (Fig. 3b). Mutation of the equivalent lysine or asparagine on TM4 and TM6 to alanine abolishes transport in *S. cerevisiae* Vrg4^24^. Using our previous repeat-swapped model of the cytoplasmic open state, we proposed that these side chains formed part of the axis of rotation around the ligand during the transport cycle^24^. Our analysis, utilising the Cryo-EM structures, now shows that while the repeat swap model was qualitatively correct in identifying the helices required for movement, the direct structural comparison reveals important subtleties within the structure during alternating access. An important aspect of this mechanism is that alternating access within Vrg4 is not symmetric, with notable differences in the helix movements on the cytoplasmic and Golgi-facing sides of the transporter. As discussed below, this asymmetry is likely to have significant consequences for ligand recognition within the wider NST family.

### Structural basis for nucleotide sugar recognition from the cytosol

From the cytoplasmic side of the membrane, Vrg4 must recognise a larger substrate, GDP-mannose, while from the Golgi lumen, the transporter captures GMP for counter-transport to the cytoplasm. Therefore, the asymmetry observed in the gating helices likely plays a significant role in substrate selection. A consequence of this structural asymmetry is the difference in size of the binding cavity between conformations in the alternating access cycle. In the inward-facing, cytoplasmic open state, the binding cavity is 21 Å wide and 24 Å deep (Fig. 2c), whereas in the opposite orientation, the cavity measures 27 Å in width and 28 Å in depth (Fig. 1e). When we overlay the structure of the GDP-mannose bound state of ScVrg4 onto the cytoplasmic open state of CaVrg4, the nucleotide sugar molecule does not fit into the cavity in the same conformation (Supplementary information Fig. 7b), implying that the nucleotide sugar must be recognised in a different orientation.

**Fig. 4:**
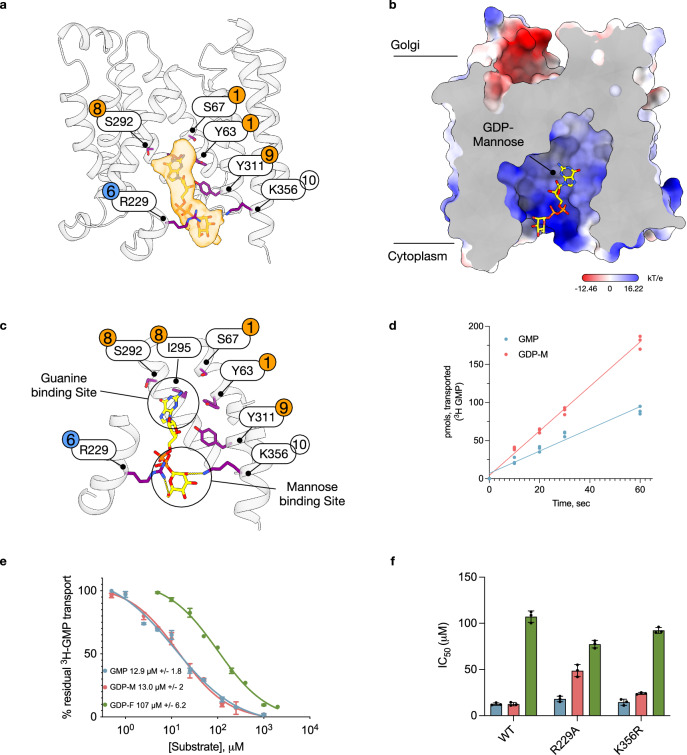
Recognition of GDP-mannose in the cytoplasmic-facing state. **a** Cryo-EM density of the GDP-mannose ligand (orange, contoured at a threshold of 0.211). Side chains sitting close (within ~ 4 Å) to the ligand are shown (purple sticks) and labelled with their respective TM helices. **b** Electrostatic surface representation of Vrg4 bound to GDP-mannose, highlighting the positively charged nature of the binding site. **c** Zoomed in view of the interactions made with the GDP-mannose. Key side chains are labelled with their respective TM helices. **d** Initial transport rates of ^3^H GMP uptake into Vrg4 liposomes, highlighting the difference when GDP-mannose is used as the antiport substrate versus GMP. (*n* = 3 independent experiments performed on different days). Each data point is shown with a line fitted through all the points (linear regression). **e** IC_50_ values for GMP, GDP mannose and GDP fucose for WT Vrg4 for these experiments. GDP-mannose was used as a counter substrate. (Values shown (mean) are calculated from three independent experiments, and errors shown are SD). **f** Calculated IC_50_ values for GMP, GDP mannose and GDP fucose for WT and mutant forms of Vrg4. (*n* = 3 independent experiments performed on different days, the mean is shown, and errors indicate SD). Source data (for **d**–**f**) are provided as a Source Data file.

Interestingly, in the maps obtained for the NB4-bound structure, we observed density consistent with the endogenous ligand, GDP-mannose (Fig. 4a, Supplementary information Fig. 8). The concentration of GDP-mannose in the yeast cytosol is very high (> 10 mM)^29^, which is likely where the ligand was captured during purification. The entrance to the binding site is positively charged, which likely facilitates capture of the GDP-mannose from the cytosol (Fig. 4b). The GDP-mannose sits in a vertical orientation with the guanine group positioned within 4 Å of many side chains that interact with the ligand in the luminal-facing state^24^. The maps obtained indicate the binding pose for GDP-mannose is highly flexible, with alternate positions for the sugar ring. Nevertheless, our analysis below is made based on the most likely position of the ligand in the binding site. In this position, the main interactions between the nucleotide sugar and the transporter include Tyr63 and Tyr311 (Tyr28 and Tyr281 in *S. cerevisiae* Vrg4), located near the nucleotide and ribose groups, respectively. Both tyrosines are critical for activity in ScVrg4^24^. Serine 67 on TM 1 is also positioned close to the nucleotide group, along with Serine 292 and 296 (Ser266) on TM 8. Serine 266 in ScVrg4 is important for nucleotide specificity, which aligns with the modelled position of GDP-mannose in the cytoplasmic-facing state. Interestingly, the two asparagines of the (F/Y)YNN motif, Asn246 and Asn247, which play an essential role in nucleotide binding in the Golgi-facing state of ScVrg4, are approximately 8 Å away from the guanine group in the cytoplasmic-facing state (Supplementary information Fig. 7c). Notably, the closest interaction between the guanine group and the binding pocket is with Ile295, which, along with the serines discussed above, is also on the gating helix TM 8. Isoleucine has been shown to play important roles in many DNA-binding proteins, forming van der Waals interactions with the nucleobase rings^30^, suggesting a similar interaction may occur within Vrg4 in the Golgi-facing state.

At the opposite end of the ligand, the mannose group interacts with Arg229 on TM6 via the C6 hydroxyl and Lys356 on TM10 via the C2 hydroxyl (Fig. 4c). In the cytoplasmic-facing state, Arg229 forms a salt bridge with Glu164 on TM4. Both TMs 6 and 4 form part of the gating helices in Vrg4 (Fig. 3). Interestingly, ScVrg4 and CaVrg4 both display altered kinetics when GMP or GDP-mannose act as the antiport substrate in liposome assays, with GDP-mannose showing faster transport rates and greater overall uptake (Fig. 4d). However, the IC_50_ values for both ligands remain similar, approximately 13 μM (Fig. 4e). If the observed density corresponds to GDP-mannose, we reasoned that mutation of Arg229 and/or Lys356 might be expected to affect the IC_50_ of GDP-mannose more than that of GMP, especially considering their interactions with the sugar group. Although a Lys356Ala mutant demonstrated poor transport properties for both ligands (Supplementary information 7d), a more conservative mutation to arginine showed transport behaviour akin to the WT protein, as did the Arg229Ala mutation (Supplementary information 7d). The IC_50_ for GDP-mannose increased to 49 μM (from 13 μM in the WT) in the Arg229Ala mutant, while its impact on GMP was minimal (18 μM versus 13 μM) (Fig. 4f). In comparison, the Lys356Arg mutation had a less significant effect, with the IC_50_ for GDP-mannose rising to 24 μM versus 14 μM for GMP. Therefore, despite the density being suboptimal for precise ligand modelling (Supplementary information Fig. 8), the transport assay results support the proposed location of GDP-mannose in the cytoplasmic-facing state.

To expand our analysis of the nucleotide binding site, we included GDP-fucose, which is not a physiological nucleotide sugar for yeast but is utilised for glycosylation in higher eukaryotes including humans^31^. Specifically, we tested whether the Arg229Ala and Lys356Arg mutants affected GDP-fucose recognition. As expected, wild-type CaVrg4 displays an eight-fold lower IC_50_ for GDP-fucose compared to GDP-mannose, indicating that recognition of the sugar moiety is important in the transport mechanism (Fig. 4e).

Interestingly, in the Arg229Ala mutant, the IC_50_ reduced from 107 μM to 77 μM. In our structure, Arg229 interacts with the hydroxyl group on the C6 carbon of the mannose moiety (Fig. 4c), suggesting that Arg229, and possibly this region on TM6, may function to recognise the sugar moiety in nucleotide sugar transporters. Indeed, in human SLC35C1 (the GDP-fucose transporter), Arg229 is replaced by a Threonine, which would facilitate fucose accommodation. However, Lys356Arg did not improve GDP-fucose recognition (Fig. 4f), despite the presence of arginine at this position in the human SLC35C1 transporter. As discussed below, we hypothesise that nucleotide sugar recognition is a dynamic process, with the ligand occupying different positions during capture and transporter across the Golgi membrane.

### Mechanism of nucleotide-sugar transport

**Fig. 5:**
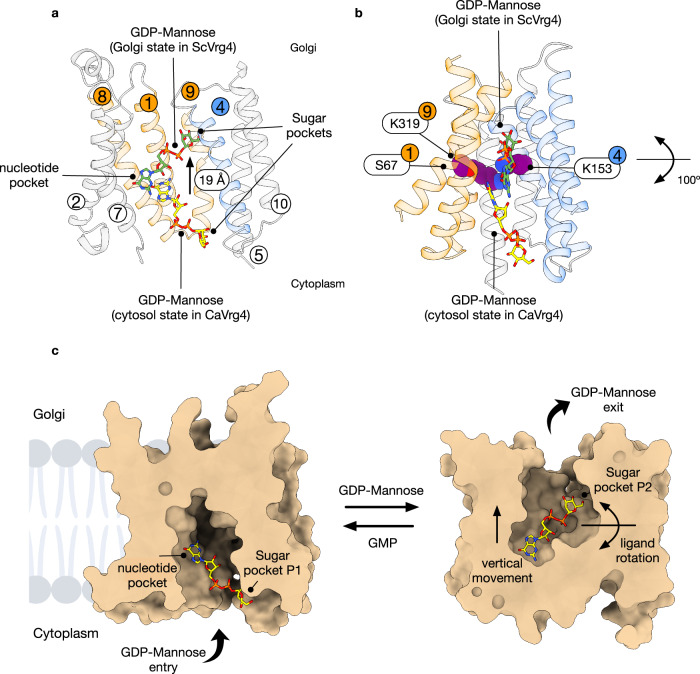
Mechanism of transport in NSTs. **a** Cartoon representation of CaVrg4 bound to GDP-mannose in the cytoplasmic facing state, with the position of the GDP-mannose from the crystal structure of ScVrg4 (PDB: 5OGK) in the Golgi-facing state superimposed. The gating helices are coloured as in Fig. 3. **b** View of the superimposed structures rotated 90° relative to panel (**a**). Side chains involved in the recognition of GDP-mannose are shown in spheres and labelled with their respective helices. **c** Model for nucleotide-sugar transport by Vrg4 with the key movements of the GDP-mannose indicated. Membrane created in BioRender. Newstead, S. (2026) https://BioRender.com/mynbro0.

Intriguingly, the position of the GDP-mannose in the cytosol-facing state of CaVrg4 differs substantially from the orientation observed in the Golgi-facing state of the ScVrg4 transporter (Fig. 5a). Indeed, the GDP-mannose undergoes an almost 100° movement between the two poses, pivoting around the nucleotide group, with the mannose group moving approximately 19 Å vertically (Fig. 5b). The axis around which the GDP-mannose moves runs through Ser67 on TM1, Lys153 on TM4 and Lys319 on TM9, which are equivalent to Ser32, Lys118 and Lys289 in ScVrg4 (Supplementary information Fig. 1f). These side chains were predicted in our repeat-swapped model of the cytosolic-facing state of Vrg4 to form key hinge regions in the alternating access mechanism, and mutations of these conserved side chains to alanine in ScVrg4 abolish transport^24,25^. Lysine 319 forms part of the conserved GALNK^319^ motif and plays an important role in sugar recognition. Thus, the GDP-mannose undergoes a rotation during transport in the protein that moves the sugar from the cytoplasmic tunnel, where it interacts with Arg229 and Lys356, all the way to the GALNK^319^ motif in the Golgi-facing state.

Together with the structural comparison discussed above, we therefore propose an updated model for alternating access transport within Vrg4 (Fig. 5c). Starting from the cytoplasmic-facing state, nucleotide sugar ligands are attracted to the transporter via electrostatic interactions, especially at the positively charged binding site (Fig. 4b). The roles of Arg229 and Lys356 at the entrance to the binding site likely promote interactions with the negatively charged nucleotide sugars. Following initial capture, the GDP-mannose ligand is first positioned through the sugar group, which may provide more specificity than the nucleotide moiety. Considering the higher concentrations of nucleotide phosphates in the cytosol, this may enable the transporter to avoid recognising nucleotide-containing metabolites.

Once correctly positioned, the GDP-mannose is drawn into the transporter through interactions between the guanine group and the conserved side chains on TMs 1, 8, and 9, which form the nucleotide pocket. The engagement of the nucleotide with these helices most likely triggers them to close around the ligand and shift inward towards TMs 4, 6, and 7. This movement pushes the nucleotide group towards the conserved (F/Y)YNN^247^ motif on TM 7, which in turn pulls the sugar group down towards the GALNK^319^ motif on TM 9 with Tyr149 on TM 4 acting to coordinate interactions with the ribose group, as observed previously in the crystal structure of the Golgi-facing state^24^. The repositioning of GDP-mannose away from Arg229 and Lys356 allows TMs 3, 4, and 6 to move more freely as the transporter transitions to the Golgi-facing state. The substantial movement of the ligand would require more extensive structural rearrangement in the transporter, which is indeed what we observed in the Golgi-facing side of the protein in our structural comparison, discussed above (Fig. 3b).

This mechanism is atypical for solute carriers, which generally hold the ligand in a fixed position and move around the ligand to transport it across the membrane^32^. However, it is qualitatively similar to the mechanism recently reported for the structurally homologous SLC35B1 ATP/ADP exchanger in the endoplasmic reticulum, which detailed the vertical repositioning of ATP during the transport cycle^20^. The movement of the GDP-mannose is also consistent with the dynamic binding mode observed for GMP in the crystal structures of the Golgi-facing state of ScVrg4^23^. The coordinated movement of both the ligand and the gating helices is also possibly a conserved feature of the DMT family^15^.

A longstanding question in the NST field has been whether a mechanism exists whereby NSTs facilitate transport direction, ensuring adequate supplies of nucleotide sugars to glycosyltransferases^7,17^. In Vrg4, we observe faster transport rates for the nucleotide sugar compared to the nucleotide monophosphate (Fig. 4d)^23^. Our proposed mechanism, in which the conjugated sugar group facilitates more efficient repositioning of the nucleotide within the binding site, results in faster transport rates for the nucleotide sugar. The necessity of a ligand to coordinate the gating helices in either the cytoplasmic to Golgi, or Golgi to cytoplasmic facing states also explains the strict antiporter behaviour seen in some members of the SLC35 family^24^.

Finally, the development of new antifungal drugs has recently slowed ^3^. Our study demonstrates that blocking nucleotide sugar transport in yeast can halt cell growth, underscoring the potential of nanobodies as therapeutic agents. However, the most likely path forward in drug development will be to identify small-molecule inhibitors. Our structures provide a foundation for structure-guided inhibitor design based on the CDR3 interactions observed in the NB3 complex, whilst also revealing a druggable pocket on the Golgi-facing side of the transporter in the NB4 complex.

## Methods

### *Candida albicans* Vrg4 protein expression and purification

The gene encoding CaVrg4 (UniProt Q5A477) was synthesised as a Gene string from GeneArt and cloned into the pDDGFP-Leu2D vector (Addgene 102334); the first 33 amino acids were excluded. Yeast Strain BJ5460 was transformed and cultivated in synthetic complete medium minus leucine (-leu) with 2% (w/v) D-glucose overnight before being diluted tenfold into -leu containing 2% (v/v) DL-lactate in a 15 L fermentation vessel (Eppendorf BioFlo 415). After 24 h, expression was induced by adding galactose (final concentration of 1%), and the cells were harvested after a further 20–24 h. Yeast cells were lysed using high pressure (38 kpsi using a Constant Systems continuous flow cell disruptor) and the membrane fraction isolated (at 235,000 *g*) following a clarification step at 30,000 *g*. The membranes were washed once in 20 mM HEPES, pH 7.5, 1 M potassium acetate, and finally resuspended in phosphate-buffered saline (PBS). Membranes were solubilised for 1 h in n-dodecyl-β-D-maltoside (DDM, Anatrace) and insoluble material was removed through centrifugation for 1 h at 235,000 *g*. 28 mM imidazole was added to the clarified lysate, and the His-tagged protein was bound to Ni-NTA resin (Thermo) in batch for 4 h at 4 °C. The resin was transferred into a gravity flow column, washed first with 8 column volumes (CV) of solubilisation buffer supplemented with 0.1% (w/v) DDM and 30 mM imidazole and then 25 CV of buffer containing 35 mM imidazole. The protein was eluted with 250 mM imidazole and dialysed overnight (20 mM Tris, pH 7.5, 150 mM NaCl, 0.02% (w/v) DDM) at 4 °C in the presence of Tobacco Etch Virus (TEV) protease. Following a reverse IMAC step, the flow through was concentrated using a 50 kDa MWCO spin concentrator (Sartoris) and applied to a Superdex 200 Increase 10/300 GL column (Cytiva) equilibrated in size exclusion buffer (20 mM Tris pH 7.5, 150 mM NaCl and 0.015 (w/v) DDM) for structural determination or in 20 mM Tris pH 7.5, 150 mM NaCl and 0.3% DM for reconstitution. An Avi-tagged (C terminus) version of the protein was purified as above and biotinylated using BirA ligase for use in Nanobody screening. Mutants were made using site-directed mutagenesis and verified via sequencing, and the protein was produced in the same way as for WT.

### Protein reconstitution into liposomes

CaVrg4 was reconstituted into liposomes using the dilution method into preformed lipid vesicles^33^. Chloroform was removed from the lipids using a rotary evaporator to obtain a thin film. The lipids were washed twice in pentane and then resuspended at 10–20 mg ml^−1^ in lipid buffer (20 mM HEPES, pH 7.5, 100 mM KCl). These lipid vesicles were frozen and thawed twice in liquid nitrogen, then stored at −80 °C until use. For reconstitution, the lipids were thawed and then extruded first through a 0.8-μm filter and then through a 0.4-μm filter. The lipid was added to purified Vrg4 in DM (at 0.5 μg μl^−1^ concentration) in batches and at a final lipid: protein ratio of 50:1 and incubated for 1 h at room temperature, then for a further 1 h on ice. After this time, the protein–lipid mix was diluted rapidly into 65 mL of assay buffer (20 mM HEPES, pH 7.5, 50 mM KCl, 2 mM MgSO_4_) and the proteoliposomes were collected through centrifugation at >200,000 x *g* for 2 h. To remove trace detergent, the proteoliposomes were dialysed overnight against a large volume (3 L) of assay buffer. After dialysis, the proteoliposomes were collected and resuspended in assay buffer to a final protein concentration of 0.5 μg μl^−1^, and then subjected to two rounds of freeze–thawing in liquid nitrogen before storage at −80 °C. The amount of protein reconstituted into the lipids was quantified by SDS–PAGE and densitometry. For immunisation, the lipid mix consisted of POPE: POPG at a 3:1 ratio, and the final lipid: protein ratio was 30:1. The final buffer was PBS. For all the assays, the lipid mix consisted of POPE:POPG:*Ecoli* Polar lipid Extract:EggPC in a 27:7:9:1 ratio.

### Nanobody selection and purification

To identify CaVrg4 specific nanobodies a library was raised through immunisation of a llama using reconstituted protein (reconstituted in POPE:POPG lipids at 3:1 ratio) as detailed in (Pardon 2014). Reconstituted material was injected intramuscularly using Gerbu LQ#3000 as the adjuvant. Immunisations and handling of the llama were performed under the authority of the project license PPL 70/8108. A 150 ml blood sample was collected, and peripheral blood mononuclear cells were prepared using Ficoll-Paque PLUS. Total RNA was extracted using TRIzolTM, and VHH cDNAs were generated by reverse transcription-PCR. Two rounds of nested PCR amplified the pool of VHH encoding sequences: firstly with ‘CALL_001’ and ‘CALL_002’, followed by ‘VHH_For’ and ‘VHH_Rev_IgG2’ and ‘VHH_Rev_IgG3’, and cloned into the SfiI sites of the phagemid vector pADL-23c. In this vector, the VHH encoding sequence is preceded by a pelB leader sequence and has a C-terminal His-Myc Tag. Electro-competent TG1 cells were transformed with the recombinant pADL-23c vector, resulting in a VHH library of about 2 × 10^8^ independent transformants. The resulting TG1 library stock was then infected with M13K07 helper phage to obtain a library of VHH-presenting phages. Phages displaying VHHs specific for CaVrg4 were enriched after five rounds of biopanning using 50 nM of biotinylated protein and capturing with Dynabeads MyOne Straptavidin T1 (Thermo Fisher). After the fifth round of panning, 95 individual phagemid clones were picked, VHH displaying phages were recovered by infection with M13K07 helper phage and tested for binding to specific Vrg4 binding by ELISA. ELISA-positive clones were sequenced, and distinct nanobodies were identified. Distinct nanobodies that appeared more than three times within the sequenced ELISA-positive hits were phylogenetically analysed, and from this analysis, 10 nanobodies, which were most distantly related, were purified on a 50 mL scale using nickel resin. Four nanobodies that expressed to the highest level in this vector and could interact with CaVrg4 via a nickel pulldown were subsequently subcloned into the pSBinit vector (Addgene 110100) using oligos (5’ ATATGCTCTTCTAGTCAGGTGCAGCTGGTCGAGTC and 5’TATAGCTCTTCATGCTGAGGAGACGGTGACCTG) and purified using nickel affinity chromatography and gel filtration. The binding properties were further analysed via ELISA, and the impact of these nanobodies on the transport efficiency of CaVrg4 was examined.

### Cryo-EM sample preparation and data acquisition

CaVrg4 post gel filtration was incubated with 1.5 molar excess of NB3 for 4 h on ice at a final Vrg4 concentration of 6 mg/ml. For the complex with NB4, this strategy results in a high proportion of uncomplexed particles; therefore, the Vrg4-NB complex was enriched by binding it to nickel resin. In brief, Vrg4 at 0.5 mg/mL was incubated with a three-fold molar excess of NB4 for 2 h on ice and then bound to nickel resin for 1 h. After extensive washing to remove uncomplexed Vrg4, the complex was eluted and dialysed against DDM size exclusion buffer.

Four microliters of CaVrg4-nb3 (4.4 mg/ml) or CaVrg4-nb4 (4.0 mg/ml) were adsorbed onto glow-discharged holey carbon-coated grids (Quantifoil 300 mesh, Au R1.2/1.3) for 10 s. Grids were blotted for 2 s at 10 °C, 100% humidity and frozen in liquid ethane using a Vitrobot Mark IV (Thermo Fisher Scientific). Movies were collected in counted mode, in Electron Event Representation (EER) format, on a CFEG-equipped Titan Krios G4 (Thermo Fisher Scientific) operating at 300 kV with a Selectris X imaging filter (Thermo Fisher Scientific) and slit width of 10 eV, at 165,000x magnification on a Falcon 4i direct detection camera (Thermo Fisher Scientific), corresponding to a calibrated pixel size of 0.732 Å. Movies were collected at a total dose of 55.0 or 53. e^−^/A^2^ (Table 1), fractionated to ~ 1.0 e^−^/Å^2^ per fraction for motion correction.

### Cryo-EM data processing

Patched motion correction, CTF parameter estimation, particle picking, extraction, and initial 2D classification were performed in SIMPLE 3.0^34^. All downstream processing was carried out in cryoSPARC^35^ or RELION 3.1^36^, using the csparc2star.py script within UCSF pyem^37^ to convert between formats. Global resolution was estimated from gold-standard Fourier shell correlations (FSCs) using the 0.143 criterion, and local resolution estimation was calculated within cryoSPARC.

The cryo-EM processing workflow for CaVrg4-nb3 is outlined in Supplementary information Fig. 2. Briefly, particles (2,647,181) were subjected to two rounds of reference-free 2D classification in cryoSPARC (1^st^ classification using 300 classes, 2^nd^ classification using 200 classes) using first a 160 Å then 140 Å soft circular mask. Selected particles (638,060) were subjected to multi-class ab initio reconstruction using a maximum resolution cutoff of 6 Å, generating four volumes. Particles (348,612) from the two most populated and structured classes were selected and non-uniform refined against one of their corresponding volumes, lowpass-filtered to 15 Å, generating a 3.3 Å map. Bayesian polishing in RELION further improved map quality to 3.1 Å after non-uniform refinement. Local CTF refinement and global CTF refinement (fitting beam tilt and trefoil) followed by non-uniform refinement yielded a 3.01 Å volume. The map was globally sharpened within cryoSPARC using the B-factor calculated from the Guinier plot.

The cryo-EM processing workflow for CaVrg4-nb4 is outlined in Supplementary information Fig. 3. Briefly, particles (8,692,632) underwent two rounds of reference-free 2D classification in cryoSPARC (1^st^ classification using 300 classes, 2^nd^ classification using 200 classes) using first a 160 Å and then 140 Å soft circular mask. Selected particles (1,074,584) were subjected to multi-class ab initio reconstruction with a maximum resolution cutoff of 6 Å, resulting in four volumes. Particles (325,091) from the most populated and structured class were selected and non-uniformly refined against one of their corresponding volumes, lowpass-filtered to 15 Å, generating a 3.3 Å map. Bayesian polishing in RELION, followed by an additional round of 2D classification (*k* = 100, with a 140 Å soft circular mask), led to the selection of 208,420 pruned particles, which were non-uniformly refined to produce a 3.2 Å volume. Local CTF refinement (fitting per-particle defocus) followed by non-uniform refinement resulted in a marginally improved 3.2 Å volume. To enhance density for GDP-mannose, alignment-free 3D classification was performed in RELION (*k* = 4, no resolution filter, regularisation parameter *T* = 40) using a soft mask focused on the transporter cavity. This produced one class containing 51,142 particles, which could be non-uniformly refined to 3.4 Å resulting in improved density for GDP-mannose. Maps were globally sharpened within cryoSPARC using the B-factor calculated from the Guinier plot or sharpened using deepEMhancer^38^.

### Model building and refinement

A model of CaVrg4, based on the ScVrg4 structure, and a nanobody structure, based on the NB from 7BC6, were docked into the globally sharpened map, adjusted where necessary by manual building using Coot v. 0.9^39^ and real-space refinement in PHENIX v. 1.21.2-5419 ^40^using secondary structure, rotamer, and Ramachandran restraints. Ligand restraints were generated using Grade2^41^. The final models were validated using MolProbity^42^ within PHENIX. Figures were prepared using UCSF ChimeraX v.1.9^43^.

### Transport assays

To analyse the effect of the nanobodies on Vrg4 activity in vitro, proteoliposomes containing CaVrg4 (or Vrg4 from *Saccharomyces cerevisiae*, or *Candida auris* as specificity controls) were incubated with 20 molar excess of nanobody. After an hour, the sample was diluted 20 times (to 500 μl), and 0.4 mM GDP-mannose was added. The liposomes were subjected to four rounds of freeze-thaw in liquid nitrogen and extruded through a 0.4-μm membrane. The liposomes were harvested through ultracentrifugation (120,000 *g* for 25 mins at 18 °C) and resuspended in assay buffer. The equivalent of 2 μg of protein was added to 50 μl of assay buffer containing 0.5 μM ^3^H-GMP (Hartmann analytic), which initiated the transport. All assays were performed at 30 °C. The uptake of radiolabeled substrate was stopped at the desired time (5 min) by rapidly filtering onto 0.22-μm filters, which were then washed with 2 × 2 mL of cold water. The amount of GMP transported inside the liposomes was calculated by scintillation counting in Ultima Gold (Revvity).

### Hygromycin B-based in vivo viability assay

For in vivo functional characterisation of the identified nanobodies the yeast NDY5 strain (MAT ura3–52a, leu2–211, vrg4–2) was transformed with an episomal vector containing either VRG4 from *C. albicans* or *S. cerevisiae*, under the control of the ADH promoter (yEP181, leucine marker) and also a yEP vector containing the NB under the control of the GAL promoter (yEP195, ura marker). An overnight culture was grown in minus -Leu-Ura medium containing 2% glucose and then diluted 1:10 into medium containing 2% lactate. After 9 h of growth, 2% galactose was added, and the cultures were left overnight. These cultures were diluted into fresh -Leu-Ura media with 2% galactose at an OD of 0.4 and split into two flasks, to one a final concentration of 0.085 mg/ml hygromycin B was added. The cultures were grown at 30 °C with shaking, and growth was monitored over the next 30 h by measuring the OD. Measurements were performed in duplicate, and the whole experiment was repeated to obtain the means shown in the figures.

### ELISA to determine K_D_

The purified nanobodies from the pSBinit vector contain a C-terminal Myc-His tag. The myc epitope was used to bind a dilution series of each nanobody to MaxiSorp plates containing protein A and anti-C-Myc antibody (Merck Cat. No. M4439). Protein A (5 mg/ml) was diluted 1:10,000 in PBS, and 100 μL was added per well. The plate was then left overnight. After washing and blocking for 30 min in (0.5% BSA in PBS), 100 μl of 1:2000 anti-c-myc antibody was added in PBS + 0.03% DDM and left for 45 min. The plates were washed, and then a dilution series of the nanobody of interest was added and left for 30 min. Following 3 × 200 μl washes, 100 μl of 50 nM biotinylated CaVrg4 (or *Cauris* Vrg4 as a control) was added for 40 min. The plates were washed before adding 100 μl of 1:5000 diluted streptavidin-peroxidase polymer, which was left for 40 min. After washing, the plates were developed using TMB (Merck T2885) and the subsequent absorbance was read at 650 nm. The background was subtracted (absorbance with no binder attached to the plate), and the absorbance of the highest binder concentration was set to 1. The K_D_ was calculated in GraphPad using the “one site – total binding” equation. The experiment was repeated in triplicate to obtain a mean and standard error.

### Reporting summary

Further information on research design is available in the Nature Portfolio Reporting Summary linked to this article.

## Supplementary information

Supplementary Information

Reporting Summary

Transparent Peer Review file

## Source data

Source Data

## Data Availability

Atomic coordinates for CaVrg4 have been deposited in the Protein Data Bank under accession codes 9S35 (NB3) [10.2210/pdb9S35/pdb], 9S36 (NB4/GDP mannose) [10.2210/pdb9S36/pdb]. The cryo-EM maps have been deposited in the Electron Microscopy Data Bank (EMDB) under accession codes EMD-54523 (NB3) and EMD-54524 (NB4/GDP-mannose). Source data are provided with this paper.
