# Supplementary material for: Structural basis for transport and inhibition of nucleotide sugar transport in pathogenic fungi
Source: Nat Commun. 2026 May 2;17:5954. doi: 10.1038/s41467-026-72729-6 (PMC13342644; doi:10.1038/s41467-026-72729-6)
Supplement: Supplementary file 1 — Supplementary Information [file 41467_2026_72729_MOESM1_ESM.pdf]

Structural basis for transport and inhibition of nucleotide sugar transport in pathogenic fungi.

Joanne L. Parker*[1,2,†], Justin C. Deme[3,†], Bjarne Feddersen[1],
Susan M. Lea*[3,4], Simon Newstead*,[1,2].

[1]Department of Biochemistry, University of Oxford, Oxford, OX1 3QU, UK; [2]The Kavli Institute for Nanoscience Discovery, University of Oxford, Oxford, OX1 3QU, UK, [3]Center for Structural Biology, Center for Cancer Research, National Cancer Institute, Frederick, MD 21702, USA. [4]Department of Structural Biology, St. Jude Children's Research Hospital, 262 Danny Thomas Place, Memphis, TN 38105-3678, USA. [†]Equal contribution

*simon.newstead@bioch.ox.ac.uk;
joanne.parker@bioch.ox.ac.uk;susan.lea@stjude.org.

Supplementary information
Figures 1-8
Tables 1-2

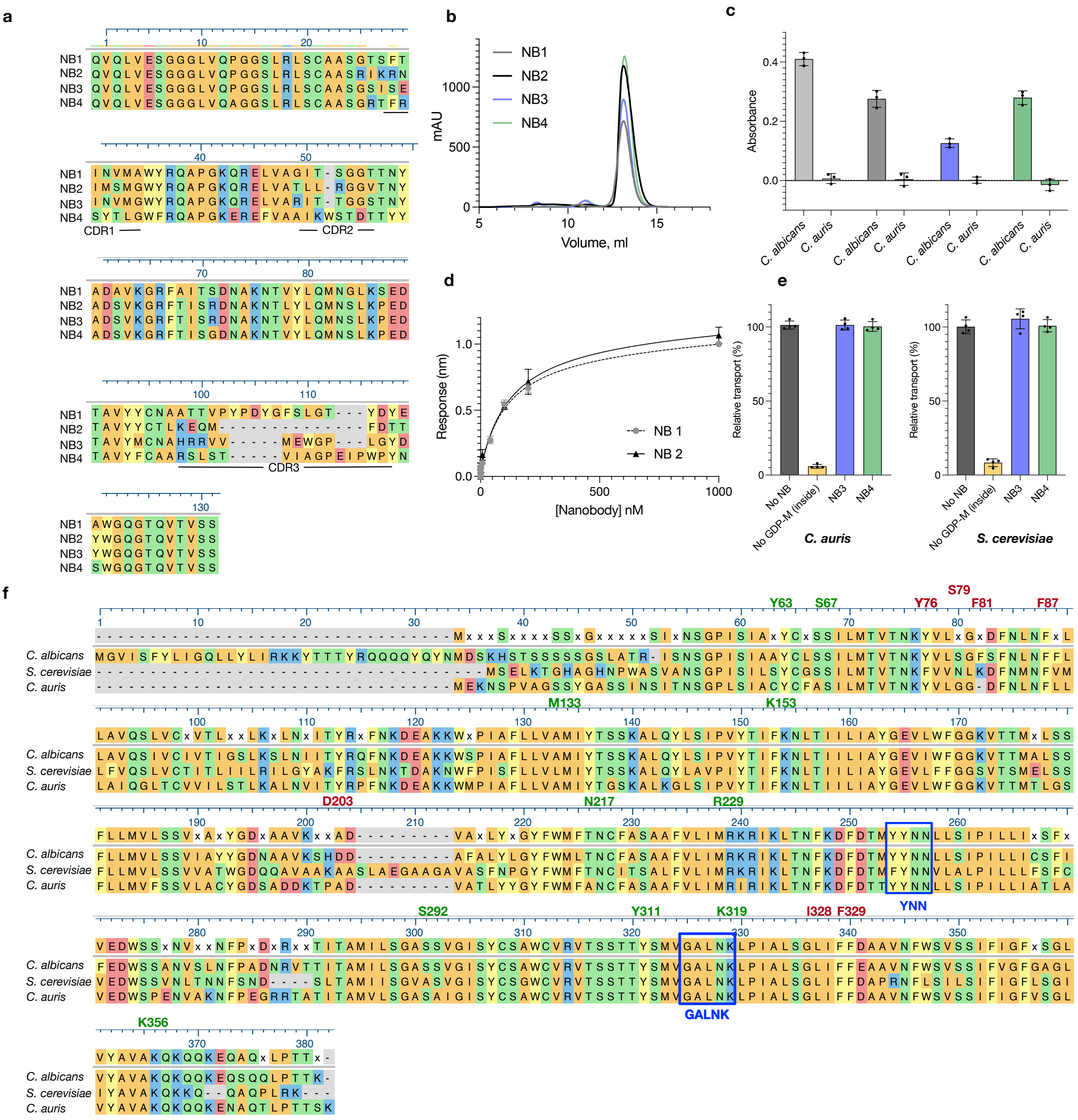

Supplementary Fig. 1. Characterisation of CaVrg4 nanobodies. a, Sequence alignment of the four nanobodies used in this study, the position of the CDR1, 2, and 3 loops is indicated. **b,** Size exclusion traces (Superdex 75) for the nanobodies used in the study. **c,** ELISA data showing the specificity of the NBs towards *C. albicans* Vrg4 as compared to Vrg4 from *C. auris*. 10 μM NB was used in the assay, and only in the presence of *C. albicans* Vrg4 was binding observed (indicated by absorbance) with no binding occurring to the *C. auris* protein. **d**, ELISA data showing normalised absorbance (normalised to the highest absorbance for each NB) against NB concentration for NB1 and NB2. The calculated K_D for both NBs was ~ 110 nM (109 ± 5 for NB1 and 125 ± 12 for NB2) (n = 3 independent experiments performed on

different days; the mean is shown, and errors indicate SD). **e**, Transport assay data showing the transport of GMP via *C. auris* & *S. cerevisiae* Vrg4 in the presence of a nanobody (NB) relative to a no NB control. Without GDP-mannose on the inside of the liposome (no GDP-M inside) this transporter cannot function. (n=4 independent experiments performed on different days, the mean is shown, and errors indicate SD). **f**, Sequence alignment of Vrg4 from *C. albicans* (Uniprot Q5A477), *C. auris* (A0A8F2VZF7) and *S. cerevisiae* (P40107). Highlighted are the conserved motifs, GALNK (sugar specificity) and YNN (nucleotide specificity). Also indicated are residues mentioned in the manuscript where NB3 or NB4 contact the transporter (shown in red) or are important for function (shown in green).

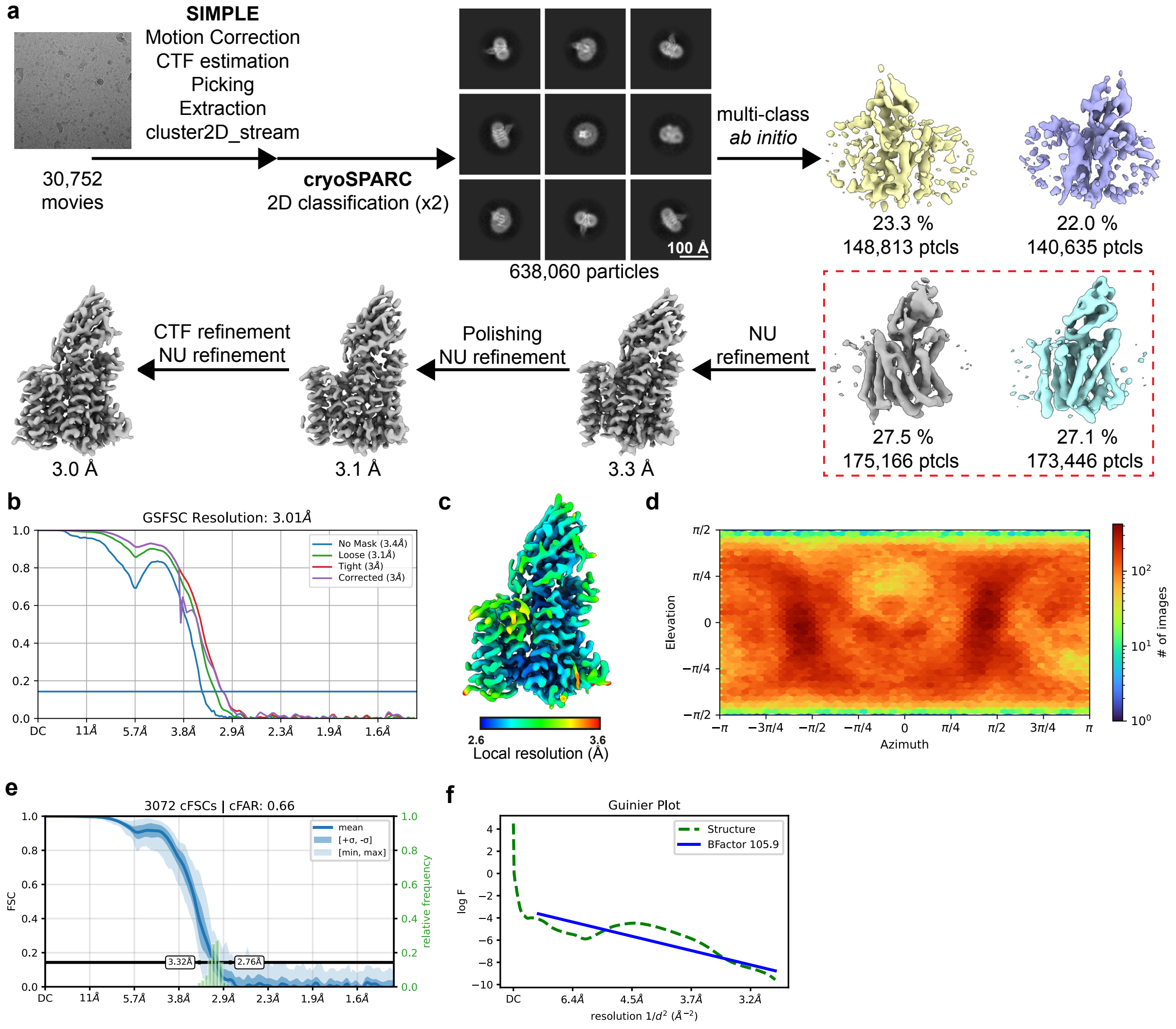

Supplementary Fig. 2 Cryo-EM processing workflow of Vrg4 with NB3. a, Image processing workflow. **b,** Gold-standard Fourier Shell Correlation (FSC) curves for global resolution. **c,** Local resolution estimate of the volume. **d,** Orientation distribution plot. **e,** cFAR score and conical FSC summary plot (calculated in cryoSPARC) for NB3 complex indicates no substantial anisotropy for this volume. **f,** Guinier plot for NB3 complex (calculated in cryoSPARC) for NB3 complex showing sharpening B factor of -105.9 Å2 applied.

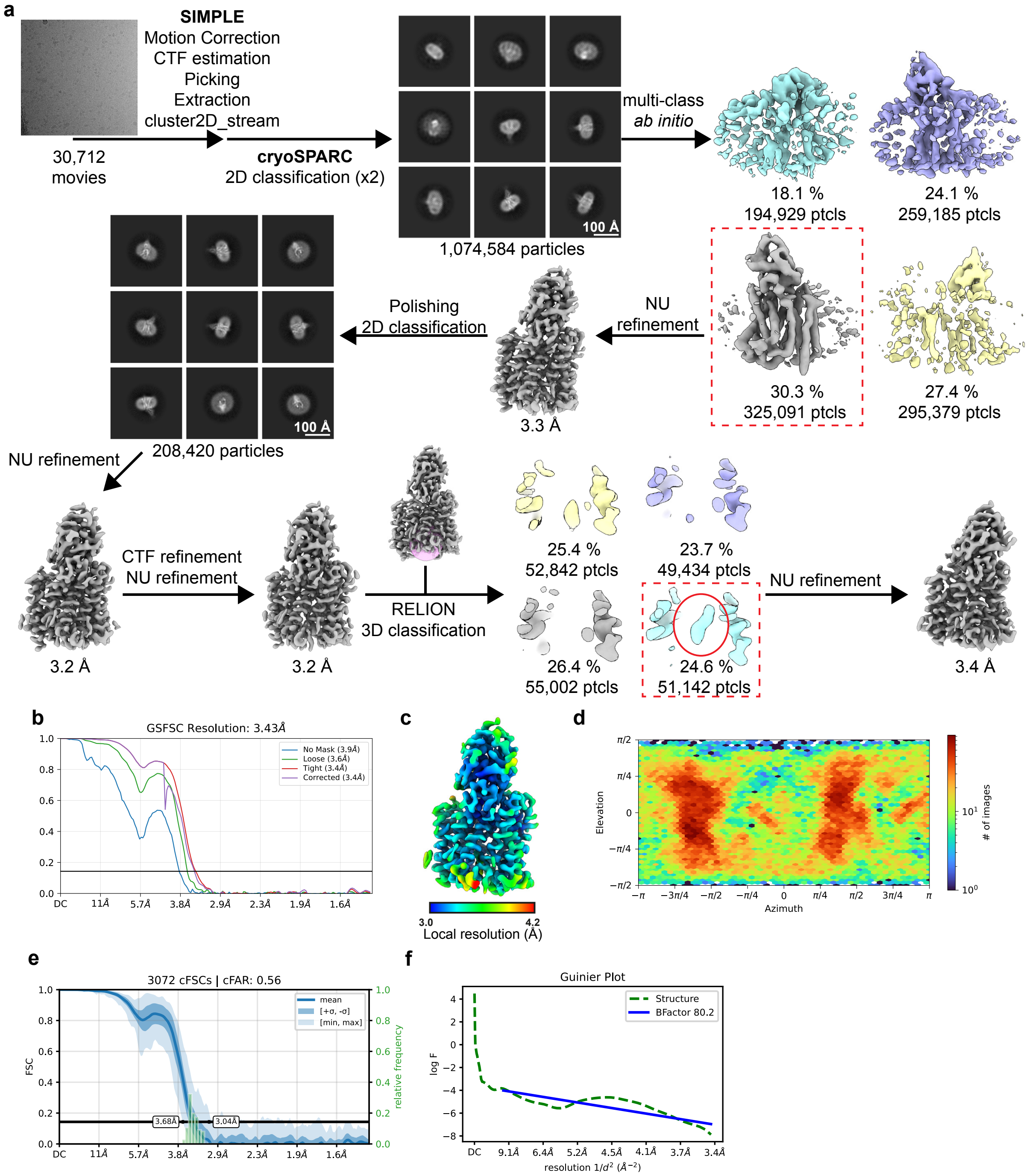

Supplementary Fig. 3 Cryo-EM processing workflow of Vrg4 with NB4. a, Image processing workflow. Volume for the endogenous GDP-mannose bound to Vrg4 is shown in the red box. **b,** Gold-standard Fourier Shell Correlation (FSC) curves for global resolution. **c,** Local resolution estimate of the volume. **d,** Orientation distribution plot. **e,** cFAR score and conical FSC summary plot (calculated in cryoSPARC) for NB4 complex indicates no substantial anisotropy for this volume. **f,** Guinier plot for NB3 complex (calculated in cryoSPARC) for NB3 complex showing sharpening B factor of -80.2 Å2 applied.

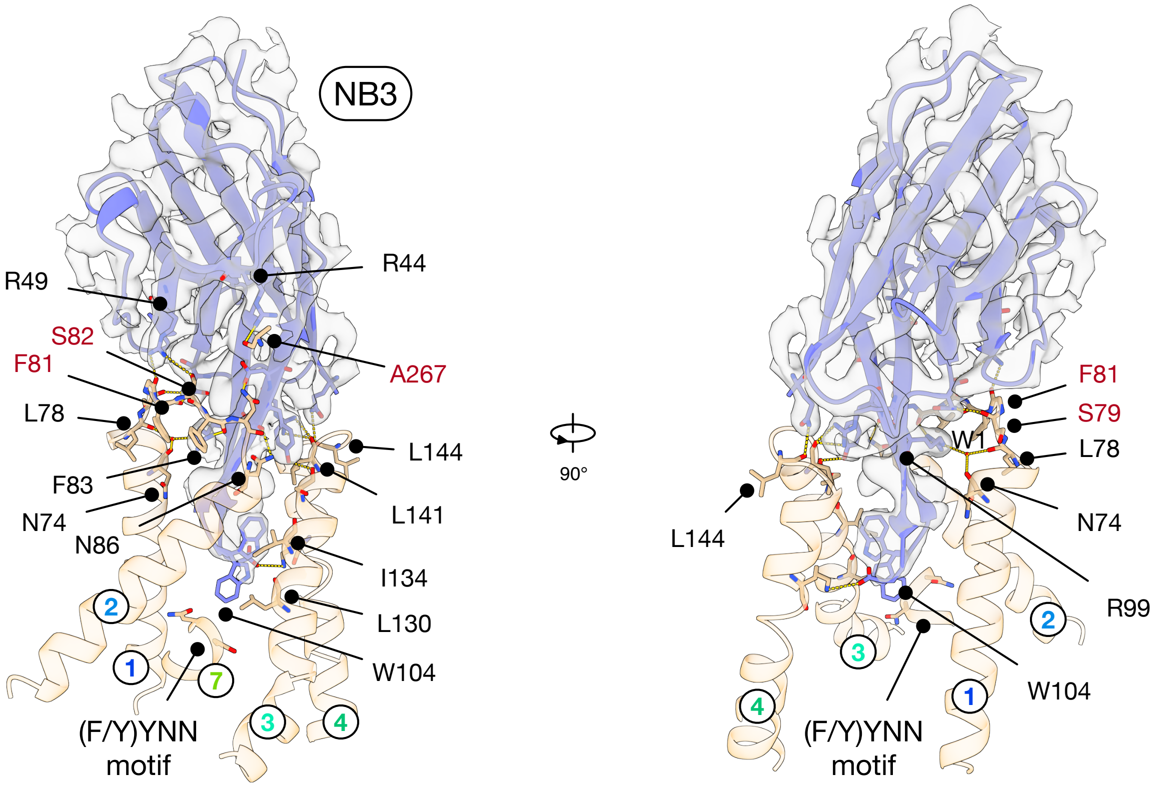

Supplementary Fig. 4. Analysis of binding interface between CaVrg4 and NB3 and NB4. Close up view of the binding interface between NB3 and CaVrg4. Side chains unique to CaVrg4 are shown in red. The cryo-EM density for NB3 is shown in grey (contoured at 0.638).

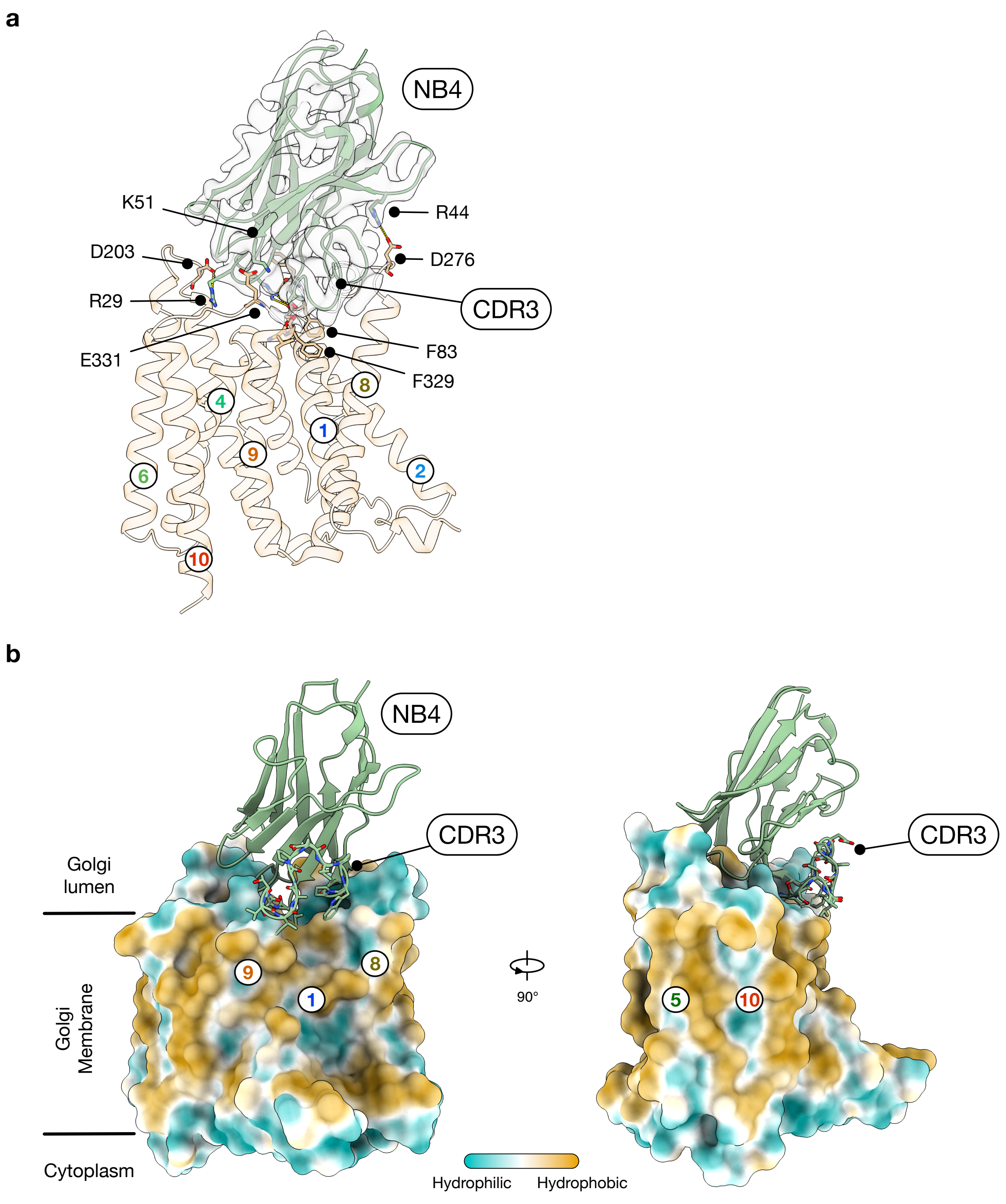

Supplementary Fig. 5. Analysis of binding interface between CaVrg4 and NB4 and NB4. a, Close up view of the binding interface between NB4 and CaVrg4. The cryo-EM density for NB4 is shown in grey (contoured at 0.534). **b,** Hydrophobic surface representation of CaVrg4 illustrating the binding position of NB4 and the unique structure adopted by the CDR3 loop to bind the cavity between TM8 and TM9 in CaVrg4.

Nanobody 3	Hydrogen Bond Dist. [Å]	Vrg4 *C. albicans*	Vrg4 *S. cerevisiae*	Vrg4 *C. auris*
Asn31 [ND2]	2.94	Ser79 [O]	Asn44	Gly47
Asn31 [ND2]	3.14	Phe81 [O]	Lys46	---
Arg44 [NH2]	3.29	Ala267 [O]	Val241	Glu234
Arg49 [NH1]	3.85	Ser82 [OG]	Asp47	Asp49
Arg49 [NH2]	2.90	Gly80 [O]	Leu45	Gly48
Arg98 [NH1]	3.07	Leu141 [O]	Leu106	Leu108
Arg98 [NH2]	3.07	Leu141 [O]	Leu106	Leu108
Arg99 [NH1]	2.92	Phe83 [O]	Phe48	Phe50
Gly108 [N]	2.85	Asn86 [OD1]	Asn51	Asn53
Tyr109 [OH]	2.80	Ser138 [O]	Ser103	Ser105
Gln0* [OE1]	3.83	Leu144 [N]	Leu109	Leu111
Glu103 [OE2]	3.73	Lys153 [NZ]	Lys118	Lys120
Asp110 [OD1]	3.16	Asn84 [ND2]	Asn49	Asn51
Arg99	Water	Leu78	Val43	Leu46
Arg99	Water	Asn74	Asn39	Asn42
	Salt Bridge Dist. [Å]			
Glu103 [OE2]	3.73	Lys153 [NZ]	Lys118	Lys120

Supplementary Table 1. Interaction analysis between Nanobody 3 and CaVrg4. PDBe PISA (https://www.ebi.ac.uk/pdbe/pisa/) interface analysis output from PDBePISA using PDB 9s35. Residues in red are unique to CaVrg4. * The first residue in the NB3 sequence is 0.

Nanobody 4	Hydrogen Bond Dist. [Å]	Vrg4 *C. albicans*	Vrg4 *S. cerevisiae*	Vrg4 *C. auris*
Arg26 [NH1]	3.40	Ser79 [O]	Asn44	Gly47
Arg26 [NH2]	3.10	Phe81 [O]	Lys46	---
Arg26 [NH2]	3.60	Phe83 [O]	Phe48	Phe50
Arg44 [NH1]	2.34	Asp276 [OD1]	Asp250	Gly243
Ser101 [OH]	2.59	Ile328 [O]	Ile298	Ile295
Ser101 [N]	2.18	Phe329 [O]	Phe299	Phe296
	Salt Bridge Dist. [Å]			
Arg29 [NE]	3.60	Asp203 [OD1]	Ala168	Asp170
Arg44 [NH2]	2.69	Asp276 [OD1]	Asp250	Gly243
Lys51 [NZ]	3.38	Glu331 [OE1]	Asp301	Asp298

Supplementary Table 2. Interaction analysis between Nanobody 4 and CaVrg4. PDBe PISA (https://www.ebi.ac.uk/pdbe/pisa/) interface analysis output from PDBePISA using PDB 9s36. Residues in red are unique to CaVrg4.

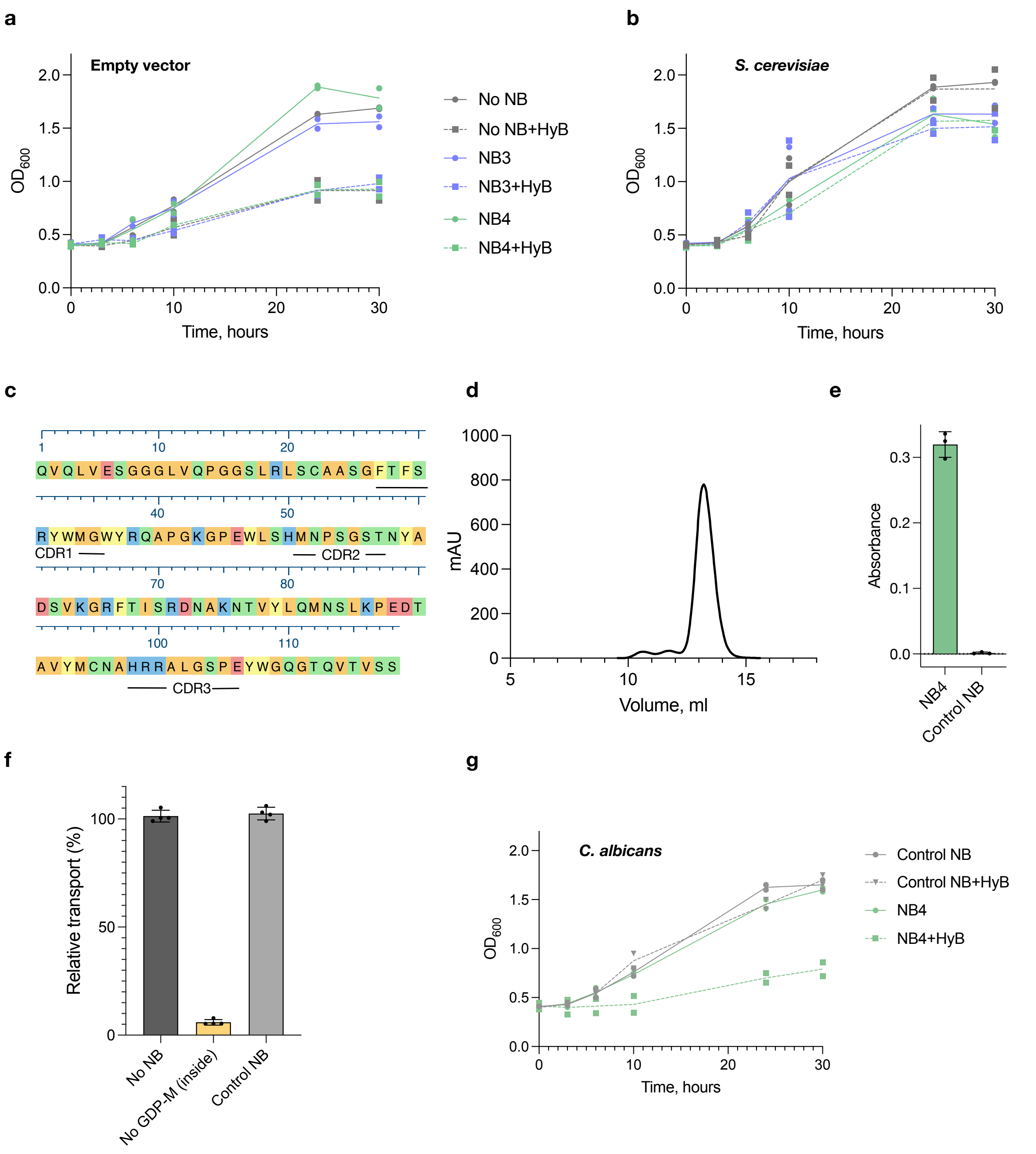

Supplementary Fig. 6. Effect of NB3 and NB4 expression on yeast strain NDY5. **a**, Growth curves of the NDY5 yeast containing an empty yEP181 vector and either NB3, NB4 or no NB in the absence or presence of hygromycin B (+HyB, dashed lines). The NDY5 strain is sensitive to hygromycin B, resulting in slower and lower growth over time; however, this sensitivity is not altered in the presence of the NBs. **b**, Growth curves of the NDY5 yeast (similar to **a**) but containing the yEP181 vector overexpressing *S. cerevisiae* VRG4 and either NB3, NB4 or no NB in the presence (+) and absence of hygromycin B (HyB). The presence of functional Vrg4 rescues the

sensitivity to HyB of this strain, which is not altered in the presence of the NBs. (for a and b, n=2 independent experiments performed on different days, all data points are shown). **c**, Sequence of a control nanobody with position of CDR1,2 and 3 indicated. **d**, Size exclusion traces (Superdex 75) for the control nanobody. **e**, ELISA data of NB4 (1 μM) and the control nanobody (20 μM) towards *C. albicans* Vrg4 showing the control NB does not bind Vrg4. (n=3 independent experiments performed on different days, the mean is shown, and errors indicate SD). **f**, Transport assay data showing the transport of GMP via *C. albicans* Vrg4 in the presence of no NB control or the control NB, indicating the control NB does not impact transport. (n=4 independent experiments performed on different days, the mean is shown, and errors indicate SD). **g**, Growth curves of the NDY5 yeast (similar to **a and b**) but containing the yEP181 vector overexpressing *C. albicans* Vrg4 and NB4 and the control nanobody in the presence (+) and absence of hygromycin B (HyB). The presence of functional Vrg4 rescues the sensitivity to HyB of this strain, which is not altered in the presence of the control NB. (n=2 independent experiments performed on different days, all data points are shown).

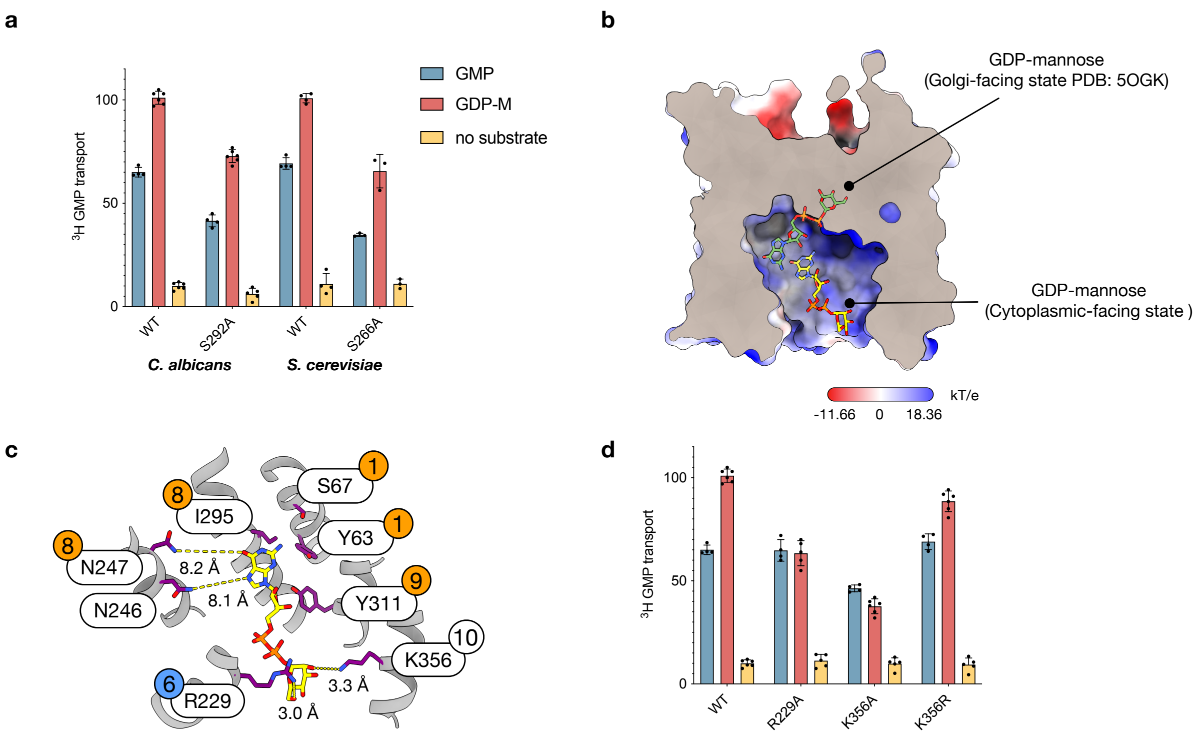

Supplementary Fig 7. Recognition of GDP-mannose in CaVrg4. a, Comparison of transport activity of WT and Ser292Ala in *C. albicans* Vrg4 and the equivalent serine (Ser266) in *S. cerevisiae*. Data is normalised to transport using GDP-mannose as the antiport substrate for the WT protein. Also shown is transport using GMP as the antiport substrate, as well as transport with no substrate, where only one turnover can occur. (n=4 independent experiments performed on different days, the mean is shown, and errors indicate SD). **b**, The position of GDP-mannose in CaVrg4 cytoplasmic facing state (shown as green sticks) with the GDP-mannose position from the Golgi facing state (PDB5:OGK, yellow sticks). **c,** Zoomed in view of the cytoplasmic facing binding site with bound GDP-mannose. Distances to key side chains are indicated. **d**, Comparison of transport activity for WT and mutant forms of CaVrg4 using either GMP, GDP mannose or no substrate as the counter substrate. Data is normalised to transport for WT with GDP mannose as the counter substrate (100%). (n=4 independent experiments performed on different days, errors shown are SD).

a

b

c

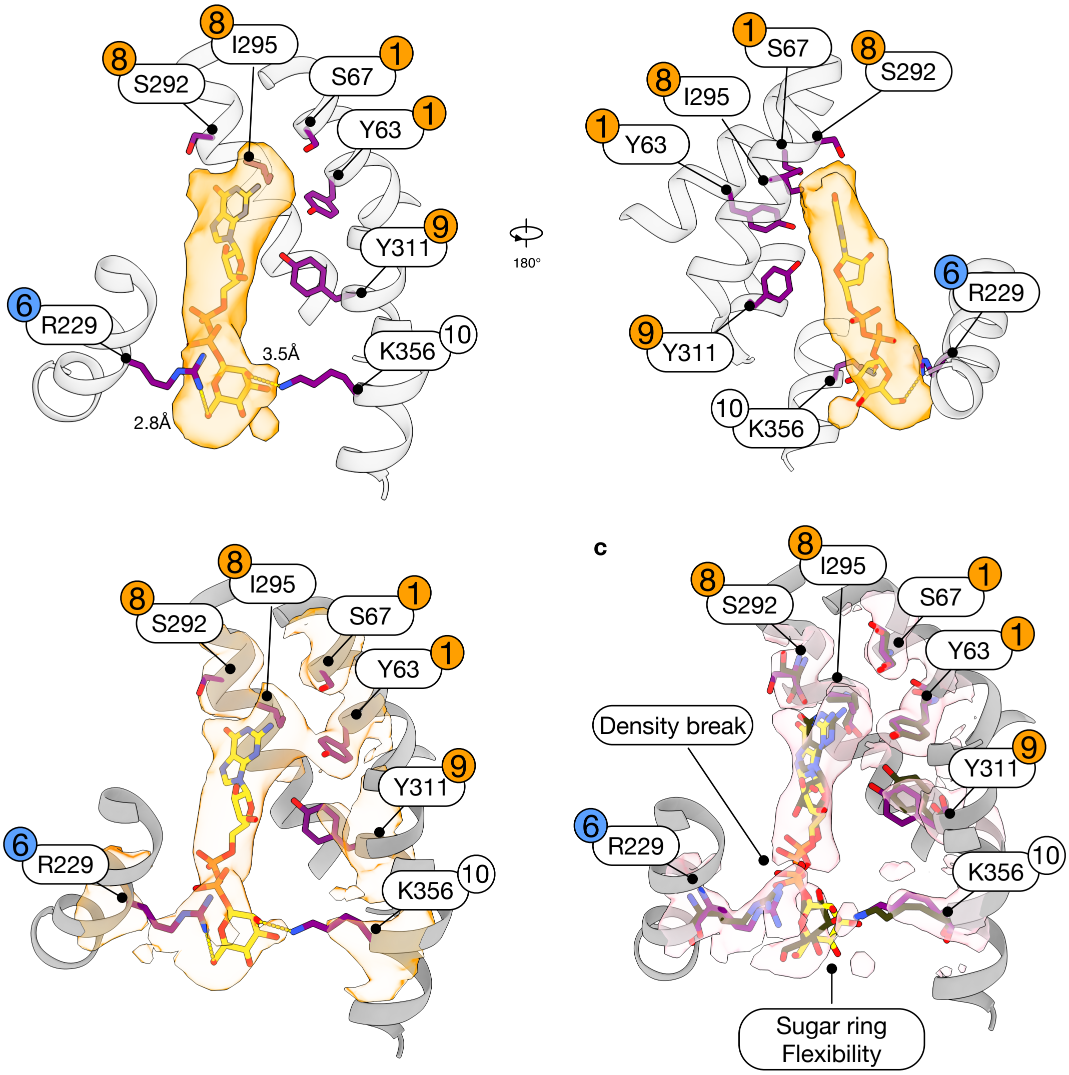

Supplementary Fig 8. Recognition of GDP-mannose in the cytoplasmic-facing state. a, Cryo-EM density of the GDP-mannose ligand (orange, unsharpened map, contoured at a threshold of 0.211). Side chains sitting close (within ~ 4 Å) to the ligand are shown (purple sticks) and labelled with their respective TM helices. Hydrogen bonds to Arg229 and Lys356 are shown and distances labelled. **b,** Cryo-EM density of the side chains modelled interacting with GDP-mannose (orange, unsharpened map, contoured at a threshold of 0.211). **c,** Cryo-EM density following B-factor sharpening (purple, contoured at a threshold of 0.461). Overlaid onto the deposited model is the alternate position of the GDP-mannose ligand (green sticks). Of note, the density in the sharpened maps is broken around the beta-phosphate, indicating increased flexibility of the sugar ring. It is possible the binding of the nanobody restricts the ability of the transporter to fully engage the substrate.
