# Supplementary material for: Structural basis for transport and inhibition of nucleotide sugar transport in pathogenic fungi
Source: Nat Commun. 2026 May 2;17:5954. doi: 10.1038/s41467-026-72729-6 (PMC13342644; doi:10.1038/s41467-026-72729-6)
Supplement: Supplementary file 3 — Transparent Peer Review file [file 41467_2026_72729_MOESM3_ESM.pdf]

nature portfolio

Peer Review File

Structural basis for transport and inhibition of nucleotide sugar transport in pathogenic fungi.

Corresponding Author: Professor Simon Newstead

This file contains all reviewer reports in order by version, followed by all author rebuttals in order by version.

Version 0:

Reviewer comments:

Reviewer #1

(Remarks to the Author)
In this study, Parker et al. report structural characterization of a GDP-Mannose transporter, Vrg4. The author captured Vrg4 in two states, with one including a putative substrate, GDP-mannose. In this study, the author used nanobody approaches to facilitate the structural determination and showed that some of those nanobodies can inhibit the transport activity both in vitro and in the fungus. Overall, the presented study is still preliminary.

The major comments are as follows:
- Since the nanobody parts are one of the major points of the paper, not just used as a fiducial mask, the presented analysis is somewhat preliminary. The binding affinity should be measured using a more standard approach, such as SPR and BLI. The monodispersity of the nanobodies on the AKTA needs to be shown. The density maps for the binding interface should be shown as well. Based on the figure, the interaction analysis is not very clear, with few specific interactions, especially for Nb3. To further support the nanobody binding mode, the interfacial mutations should be introduced into the nanobodies to disrupt binding and test their effects on the binding or the transport inhibition (IC50 of the nanobodies).

- The author claimed that the inhibitory nanobodies work in the organism. More evidence should be provided, not just a growth assay. For the control, an isotype nanobody should be used, instead of “no nanobody”. Since the affinity and IC50 of the nanobody to the transporter were not characterized very well, it’s hard to judge what’s going on. Nanobodies might simply affect the localization of the transporter through weak interactions, instead of a direct inhibition of the activity. Imaging or Glycosylation defects on the glycoproteins can be measured to gain a better understanding.

- The conformational switch should be analyzed in a better way, and the key residues involved in the conformational switch can be pinpointed, and their effects on the transport activity can be tested.

- The transport activity was not done in a sophisticated way. Since a proteoliposome-based assay was used, the kinetics of the transport and Vmax/Km should be measured, either with or without nanobodies. Similar assays should be done for all mutants.

- Regarding the claimed substrate, it is supposed to be the highlight of the paper, but actually, it is the major caveat. The author tried a focused 3D classification to get a better and more continuous density for the ligand. However, based on the resolved map, the modeling of the substrate seems quite challenging, and I think the model for the substrate is very likely to be wrong. The guanine and ribose should not be in their current positions. Therefore, more evidence needs to be provided to support the model.

- Improving the density map for the ligand will address most of the issues. Maybe more data can be collected, and classification using more classes can be done. Currently, the particle recovery rate is quite low (only 11 particles per image). Maybe a nanobody-enlargement approach, such as Fab/megabody/legobody, can be used to increase alignment accuracy and then particle yield. Based on the decline of FSC between 3.8~7A (ideally, it should be close to 1), the alignment accuracy is not good, which will affect the following 3D classification without alignment. In the Extended Figure 3, why was the class in yellow abandoned? Density subtraction might be used to improve classification efficiency. The root problem for the sub-optimal substrate density could be that the nanobody selection was done using the apo form; therefore, the conformation might not be optimal for ligand binding, and the nanobody selected could be even in competition with the

ligand for a conformational switch. Therefore, the protein sample with ligand bound could be used to select for a better nanobody through in vitro screening.

- With a better density map, a better interaction mode with different interacting residues can be tested, using a more sophisticated way of assaying.

Reviewer #2

(Remarks to the Author)
This is a very well-written manuscript by authors who have been studying NSTs for a number of years.

The overall premise that this will aid drug discovery is doubtful; however, the work is worth publishing. The dynamics of the transition between the cytoplasmic open form and the Golgi open form are very well described. Interestingly, all earlier research on these dynamics assumed symmetric movements, but this clearly shows that the movement is not symmetric. The data is convincing. However, I am surprised that the authors have not utilized the two-endpoint structure (with the cytoplasmic-bound GDP-Mannose) to perform a simple targeted MD simulation, which could reveal a lot about the transport mechanism's dynamics.

I was also disappointed that the authors did not fully explore the sugar specificity argument. They mutate Arg 229 and Lys 356 to demonstrate their roles in recognizing GDP mannose. However, they do not test whether altering these residues affects sugar specificity. Although excellent biochemical data is available, I assume the authors mutated these residues to change sugar specificity and did not observe transport of other GDP sugars. If so, then some of their arguments about specificity are incorrect. I suggest this not because these residues aren't important, but because the dynamics and flipping of the sugar involve more residues, some of which may also contribute to specificity.

Reviewer #3

(Remarks to the Author)
In the present manuscript, Parker et al. report two cryo-EM structures of the GDP-mannose transporter Vrg4 from Candida albicans, each bound to a different nanobody. The nanobodies stabilize distinct conformations of the transporter, yielding structures that represent the cytoplasmic and Golgi-facing states. These findings provide valuable insights into the conformational changes that occur during the transport cycle of this solute carrier family. Moreover, the authors identified GDP-mannose as an endogenously bound ligand in one of the structures, enabling them to draw conclusions about ligand recognition and translocation during transport. The structural work is complemented by biochemical transport assays and mutational studies.
Overall, this is a well-executed study that offers novel insights into the structure, function, and conformational dynamics of the SLC35 transporter family. The structural and biochemical analyses are of high quality, and the authors' conclusions are well supported by the data. The figures are clear and effectively illustrate the findings.
I have one general comment and several specific remarks that should help improve the quality of the manuscript.

General comment:
1) The authors present a structure of Vrg4 bound to GDP-mannose, which appears to have been co-purified with the protein. A comparison between the apo and ligand-bound forms of the same conformational state would be highly informative, as it would allow structural changes in the binding site to be monitored in the absence versus presence of the ligand. In the Methods section, the authors note that they improved the ligand density through 3D classification, which suggests that the "unused" classes might correspond to an apo form — depending on the effectiveness of the classification. If feasible, it would be valuable for the authors to attempt a reconstruction of the apo form from their existing data and to compare it with the ligand-bound structure.

Specific comments:
a) Table 1 is a bit minimalistic; it should include Ramachandran statistics and Map sharpening B factors (Å2)
b) No cryo-EM volume for any of the determined structures (only PDBs and a surface representation of the PDB) is shown in the main figures. Please add this information as a panel to the figures.
c) The authors should include a directional FSC diagram in the processing figures as well as cFAR values.
d) A Ligplot-style diagram highlighting the interactions between the ligand and sidechains of the transporter should be added. This will make it easier for the reader to spot crucial interactions.

Reviewer #4

(Remarks to the Author)
I co-reviewed this manuscript with one of the reviewers who provided the listed reports. This is part of the Nature Communications initiative to facilitate training in peer review and to provide appropriate recognition for Early Career Researchers who co-review manuscripts.

Version 1:

Reviewer comments:

Reviewer #1

(Remarks to the Author)
In the revised version, Parker et al. added more detailed illustrations of the interaction of the nanobody with Vrg4 and presented more experiments characterizing the nanobody. Moreover, they argued that IC50 and ELISA are as robust as more sophisticated assays, which is fine. They have also made an intensive effort to provide better support for the modeling of the substrate.

The issue with the substrate seems not only to be the occupancy problem, but also flexibility. I still find the binding pose of the substrate quite weird in terms of its interaction with protein side chains, and I reserved my concerns about the accuracy of the modeling of the substrate. Mutagenesis only pinpoints protein residues important for substrate binding, but doesn’t provide information on the binding pose of the substrate. It would be helpful for potential readers to understand the current model better by showing the cryo-EM density of the interacting residues, along with the substrate density (it should be a routine presentation). And MD simulation might be useful to test the stability of the current model.

If everyone else thinks the current substrate binding pose is fine, then go ahead with publication.

Reviewer #2

(Remarks to the Author)
The authors have sufficiently responded to all of my comments. I support the manuscript for publication.

Reviewer #3

(Remarks to the Author)
The authors have satisfactorily answered my questions and considered different suggestions. I recommend the paper for publication.

Reviewer #4

(Remarks to the Author)
I co-reviewed this manuscript with one of the reviewers who provided the listed reports. This is part of the Nature Communications initiative to facilitate training in peer review and to provide appropriate recognition for Early Career Researchers who co-review manuscripts.

Open Access This Peer Review File is licensed under a Creative Commons Attribution 4.0 International License, which permits use, sharing, adaptation, distribution and reproduction in any medium or format, as long as you give appropriate credit to the original author(s) and the source, provide a link to the Creative Commons license, and indicate if changes were made.

In cases where reviewers are anonymous, credit should be given to 'Anonymous Referee' and the source.
The images or other third party material in this Peer Review File are included in the article's Creative Commons license, unless indicated otherwise in a credit line to the material. If material is not included in the article's Creative Commons license and your intended use is not permitted by statutory regulation or exceeds the permitted use, you will need to obtain permission directly from the copyright holder.
To view a copy of this license, visit https://creativecommons.org/licenses/by/4.0/

RESPONSE TO REVIEWER COMMENTS.

We would like to thank all the reviewers for taking the time and effort and to read and comment on our manuscript. We appreciated the considerable effort these reviews take and the increasing burden on researchers at all stages of their career. We have taken on board the comments and revised our paper accordingly. Thank you.

Reviewer #1 (Remarks to the Author):

In this study, Parker et al. report structural characterization of a GDP-Mannose transporter, Vrg4. The author captured Vrg4 in two states, with one including a putative substrate, GDP-mannose. In this study, the author used nanobody approaches to facilitate the structural determination and showed that some of those nanobodies can inhibit the transport activity both in vitro and in the fungus.

The major comments are as follows:
- Since the nanobody parts are one of the major points of the paper, not just used as a fiducial mask, the presented analysis is somewhat preliminary. The binding affinity should be measured using a more standard approach, such as SPR and BLI.

- While we appreciate the reviewer's suggestion, we opted to use the ELISA method for this study because we have found it to be robust and ideal for assessing and ranking a library of nanobodies against a target protein. ELISA has long been the primary technique for determining antibody binding affinity. We specifically chose ELISA because it is highly sensitive, very specific, and cost-effective. In our extensive experience of generating nanobodies against solute carrier proteins, we have found ELISA to be just as, if not more, reliable than BLI or SPR, which are generally more suited to monoclonal antibody analysis. We also aimed to highlight this point in our study, as many early career researchers do not have access to high-end BLI and SPR instruments and may not realise they can conduct equally useful binding studies using simple ELISA. Using an alternative method would only recalculate a KD and would not alter the conclusions of our paper. We acknowledge that determining on and off rates for the nanobodies could be informative for dissecting the contributions of individual interactions, as would ITC. However, we believe these detailed calculations would not significantly change the core message of this section, which shows that i. nanobodies can be generated against nucleotide sugar transporters, ii. when expressed in yeast cells, they arrest transporter function, and iii. Inhibition of Vrg4 leads to growth arrest of pathogenic fungi.

The monodispersity of the nanobodies on the AKTA needs to be shown.

- We apologise for not including this data in the original version. We have now included this data in Extended Data Figure 1b.

The density maps for the binding interface should be shown as well.

- We agree, and we have now included the figures illustrating the binding interface in Extended Data Figures 4 and 5, along with a considerably more in-depth analysis of the binding interface.

Based on the figure, the interaction analysis is not very clear, with few specific interactions, especially for Nb3. To further support the nanobody binding mode, the interfacial mutations should be introduced into the nanobodies to disrupt binding and test their effects on the binding or the transport inhibition (IC_{50} of the nanobodies).

- We apologise if our initial figures were unclear. We have now considerably revised this section of the paper by i. Including a larger view of the interfaces between CaVrg4 and NB3 in Figure

1 and Extended Data Figure 4 and for NB4 in Figure 2 and Extended Data Figure 5. In addition, we have included the PDBe PISA analysis in Extended Data Tables 1 and 2. As we illustrate and detail in Extended Data Table 1, NB3 forms eight interactions within the binding site, and species specificity is driven by interactions on the loops that form the transporter's outer surface. We have shown these as red in the figures and table for clarity. We have clearly shown that both nanobodies are specific for *Candida albicans* Vrg4 over both a distant *(S. cerevisiae*) and a close homolog (*C. auris*), both *in vitro* and *in vivo*. We have included the equivalent side chains in Extended Data Tables 1 and 2 to illustrate this point, which combined with the sequence alignment in Extended Data Figure 1 should be sufficient for the reader to understand where the specificity originates in CaVrg4 recognition. We thank the referee for prompting us to make these changes as we think this is an important aspect that was initially under discussed.

The author claimed that the inhibitory nanobodies work in the organism. More evidence should be provided, not just a growth assay. For the control, an isotype nanobody should be used, instead of "no nanobody". Since the affinity and IC_{50} of the nanobody to the transporter were not characterized very well, it's hard to judge what's going on.

- The reviewer's suggested control, using an isotype-specific nanobody, has now been included in Extended Data Figure 6e-g, and the sequence has been added as an additional data file 1. The control nanobody shows the same effect as the 'no-nanobody' control, which also addresses the question raised in the preceding comment regarding mutating the CDR1-3 regions (as this nanobody has different side chains in these regions of the otherwise identical nanobody scaffold). In the presence of *C. albicans* Vrg4, the NDY5 yeast strain grows equally with or without hygromycin B. These data support the original conclusion that the presence of either NB3 or NB4 inhibits CaVrg4 in yeast cells, leading to growth arrest.

Nanobodies might simply affect the localization of the transporter through weak interactions, instead of a direct inhibition of the activity. Imaging or Glycosylation defects on the glycoproteins can be measured to gain a better understanding.

- Our logic is that the nanobodies were raised against protein reconstituted into a lipid environment, and we demonstrate these nanobodies bind to and inhibit transport in liposomes (Figure 1a-b). Subsequently, we demonstrate that in yeast cells, these same nanobodies result in loss of function (Figure 2f). The growth assay we used depends on an active form of Vrg4; therefore, we conclude that the nanobodies have disrupted the transporter's function. Although we cannot rule out that nanobody binding affects the transporter's localisation (rather than its function), the conclusion remains the same: the nanobody prevents Vrg4 from functioning in the cell. Given the structural data, which clearly shows interactions between the unique CDR1-3 regions of both NB3 and NB4 to the transporter, and the lack of binding of an isotype nanobody (new data) coupled with the strength of the interaction, we conclude that the most likely explanation is direct inhibition in the cell. The referee makes a reasonable point regarding the assessment of glycosylation defects or indeed localisation. If we were working with mammalian cells, then we would agree. However, in yeast cells determining mislocalisation between the ER and Golgi is very difficult (if not impossible given the size of the Golgi apparatus) as would quantitative analysis of the glycosylation defects. We would therefore argue that while these suggestions are very suitable for mammalian cell studies, in this instance they would expend considerable time and funds without changing the conclusion, that expressing NB3 and NB4 in the yeast cells arrest their growth in the presence in HygromycinB (which is known to kill yeast cells with defective mannosylation).

The conformational switch should be analyzed in a better way, and the key residues involved in the conformational switch can be pinpointed, and their effects on the transport activity can be tested.

- The conformational differences between the helices are shown in Figures 3 & 4 and colour-coded to highlight the significant changes between the two conformations. The side chains we identify as being critical for the conformational changes are discussed in the section 'Structural comparison between the Golgi and cytosol-facing states' and 'Mechanism of nucleotide-sugar transport'. We also specifically mention the side chains in lines 205-207, 218-219, 273, and 278, and include them in the sequence alignment in Extended Data Fig. 1. We also hope that interested readers will refer back to our other papers on this protein[1-3] on which this section of the paper builds and expands.

The transport activity was not done in a sophisticated way. Since a proteoliposome-based assay was used, the kinetics of the transport and Vmax/Km should be measured, either with or without nanobodies. Similar assays should be done for all mutants.

- Rather than determining a Km or Vmax we chose instead to use IC_{50} data to understand the role of specific side chains observed in structures that contact the GDP-mannose ligand, thus providing mechanistic details on ligand selection. The only side chain we did not include in our previous studies[1-3] was Ser292 (Ser266 in *S. cerevisiae* Vrg4), which was why we undertook these assays and included the data in Extended Data Fig. 7a. The remaining side chains we identified were all previously characterised in our earlier papers[1-3]. The structural comparison we undertook in this study represents the most extensive to date for a member of the SLC35 transporter family and provides genuinely new insights into the asymmetric alternating access mechanism within the NST family. We expect that asymmetric mechanisms are more common than many in the field have realised, so this study makes an important and substantial contribution to the mechanism of membrane transport.

Regarding the claimed substrate, it is supposed to be the highlight of the paper, but actually, it is the major caveat. The author tried a focused 3D classification to get a better and more continuous density for the ligand. However, based on the resolved map, the modeling of the substrate seems quite challenging, and I think the model for the substrate is very likely to be wrong. The guanine and ribose should not be in their current positions. Therefore, more evidence needs to be provided to support the model.

- We appreciate that modelling dynamic ligands in small membrane proteins is currently at the cutting edge of mechanistic structural studies in this field. The reviewer is right to raise this challenge, and it is one that we take very seriously and employ many different methods to validate our modelling and structure building. For example, we have discovered that ligand density is impacted very differently by sharpening of different types (global application of a B-factor, deepEMhancement etc.). We have now included an additional Extended Data Figure 8 that shows multiple-angle views of the ligand within the volume, allowing the reader a better appreciation of the information that drove our docking. We would also like to highlight that we never rely on the EM maps alone to drive our docking decisions. Indeed, in all our studies, as with this one, we use extensive mutagenesis to test the binding poses and use all the information to make our final decisions. In the case of this structure, we made several mutations to the side chains that interact with the sugar group in GDP-mannose

(Arg229 and Lys356) and demonstrate these do impact nucleotide sugar recognition (GDP-mannose vs. GDP-fucose) in Figure 4f.

Improving the density map for the ligand will address most of the issues. Maybe more data can be collected, and classification using more classes can be done. Currently, the particle recovery rate is quite low (only 11 particles per image). Maybe a nanobody-enlargement approach, such as Fab/megabody/legobody, can be used to increase alignment accuracy and then particle yield. Based on the decline of FSC between 3.8~7A (ideally, it should be close to 1), the alignment accuracy is not good, which will affect the following 3D classification without alignment. In the Extended Figure 3, why was the class in yellow abandoned? Density subtraction might be used to improve classification efficiency. The root problem for the sub-optimal substrate density could be that the nanobody selection was done using the apo form; therefore, the conformation might not be optimal for ligand binding, and the nanobody selected could be even in competition with the ligand for a conformational switch. Therefore, the protein sample with ligand bound could be used to select for a better nanobody through in vitro screening.

- We agree with the reviewer that we would always want to interpret the highest resolution version of any volume. However, our now significant experience with solving the structures of small membrane proteins, and the amount of data (in excess of 30,000 movies for both datasets) already incorporated in this study, mean that we do not think there is any reason to think that small experimental modifications will yield substantially higher resolution volumes. The reviewer comments on the small number of particles used in the final volume c.f. the number collected – we note that the percentage is entirely typical of other structures of small membrane proteins. Several of the approaches suggested by the reviewer work well for larger complexes and when the volume focused on is a larger percentage of the whole volume (e.g. density subtraction), but these approaches are of less utility where the object being aligned is so small. 3D classes were abandoned when inclusion of the particles contributing to those classes did not improve the volume (as is standard in the field) and the workflows, of course, do not summarize the many failed attempts to generate higher resolution as they only highlight the paths to the volumes used in preparation of the manuscript (again as is standard in the field). At the end of the day, the volumes obtained are sufficient to allow us to correctly deduce the ligand orientation and we therefore decided there was no justification in either collecting further data from these samples or in going back to the laborious regeneration of other nanobodies or similar open-ended experimental approaches.

"Maybe a nanobody-enlargement approach, such as Fab/megabody/legobody, can be used to increase alignment accuracy and then particle yield."

- We do appreciate the reviewer's suggestion, and we have used this method in the past. However, we do not feel that particle alignment was the limiting factor for this volume. Particle alignment was accurate enough to yield a ~3.2 Å volume before 3D classification. We have determined many nanobody-bound, ligand-bound membrane protein structures previously (PCFT, PepT2, etc). We believe the issue with the weaker GDP-man ligand density is to do with occupancy.

"Based on the decline of FSC between 3.8~7A (ideally, it should be close to 1), the alignment accuracy is not good, which will affect the following 3D classification without alignment".

- For large soluble protein complexes solved to high resolution the FSC is typically close to 1 at 3.8 – 7 Å resolution. The FSC dip at 5.7 Å is very typical for membrane proteins in detergent/nanodisc environments, likely driven by the alignment of detergents/lipids.

"In the Extended Figure 3, why was the class in yellow abandoned?"

- The "ligand" density for this class was much ropier than the blue class. Possibly believed the yellow density could correspond to a loop/terminus – in any case the reconstruction from this class was poor.

Density subtraction might be used to improve classification efficiency.

- In our experience, density subtraction of small proteins does not improve classification efficiency. Multiple classification strategies were tested – here we show the strategy that led to interpretable ligand density.

Reviewer #2 (Remarks to the Author):

This is a very well-written manuscript by authors who have been studying NSTs for a number of years.

- We thank the referee for their supportive comments.

The overall premise that this will aid drug discovery is doubtful; however, the work is worth publishing. The dynamics of the transition between the cytoplasmic open form and the Golgi open form are very well described. Interestingly, all earlier research on these dynamics assumed symmetric movements, but this clearly shows that the movement is not symmetric. The data is convincing. However, I am surprised that the authors have not utilized the two-endpoint structure (with the cytoplasmic-bound GDP-Mannose) to perform a simple targeted MD simulation, which could reveal a lot about the transport mechanism's dynamics.

- We did discuss this possibility with our Molecular Dynamics colleagues. However, as multiple conformations of both ligands are observed (this work and our previous study on the GMP ligand[2]), a study to address the complete substrate-driven conformational cycle would require detailed analysis, starting with atomistic calculation of all potential ligand binding sites as well and then combining this with the analysis of the conformational dynamic landscape. We agree with the reviewer that this could reveal much about this transporter. Still, we have to agree with our colleagues that this level of analysis and the work involved would warrant a separate study.

I was also disappointed that the authors did not fully explore the sugar specificity argument. They mutate Arg 229 and Lys 356 to demonstrate their roles in recognizing GDP mannose. However, they do not test whether altering these residues affects sugar specificity. Although excellent biochemical data is available, I assume the authors mutated these residues to change sugar specificity and did not observe transport of other GDP sugars. If so, then some of their arguments about specificity are incorrect. I suggest this not because these residues aren't important, but because the dynamics and flipping of the sugar involve more residues, some of which may also contribute to specificity.

- We apologise for this oversight, which has now been rectified. In our first version of the paper, we observed the proximity of the mannose sugar group to Arg229 and Lys356 at the mouth of the cytoplasmic tunnel. We showed, using liposome-based IC50 data, that both Arg229 and Lys356 are important for ligand selectivity, particularly for the sugar moiety. To address the reviewers' comments, we have now expanded our analysis to include GDP-fucose, which is not a physiological nucleotide sugar for yeast but is utilised for glycosylation in higher eukaryotes. This new data is now shown in Figures 4e and 4f. In brief, the recognition of GDP-fucose is ~ 8-fold less efficient than either GDP-mannose or GMP, which are the physiological substrates (Fig. 4e). Interestingly, Lys356 is an arginine in the human GDP-fucose transporters (SLC35C1). However, within Vrg4 homologues, this side chain does not impact the recognition of GDP-fucose. However, the Arg229Ala mutant showed increased affinity for GDP-fucose, supporting our structural hypothesis that ligand recognition and transport are dynamic, with multiple binding poses contributing to the transport cycle (hence the challenge with using MD as discussed above). We have added this data into lines 323-335.

Reviewer #3 (Remarks to the Author):

In the present manuscript, Parker et al. report two cryo-EM structures of the GDP-mannose transporter Vrg4 from Candida albicans, each bound to a different nanobody. The nanobodies stabilize distinct conformations of the transporter, yielding structures that represent the cytoplasmic and Golgi-facing states. These findings provide valuable insights into the conformational changes that occur during the transport cycle of this solute carrier family. Moreover, the authors identified GDP-mannose as an endogenously bound ligand in one of the structures, enabling them to draw conclusions about ligand recognition and translocation during transport. The structural work is complemented by biochemical transport assays and mutational studies.

Overall, this is a well-executed study that offers novel insights into the structure, function, and conformational dynamics of the SLC35 transporter family. The structural and biochemical analyses are of high quality, and the authors' conclusions are well supported by the data. The figures are clear and effectively illustrate the findings.
I have one general comment and several specific remarks that should help improve the quality of the manuscript.

- We thank the referee for their supportive comments.

General comment:
1) The authors present a structure of Vrg4 bound to GDP-mannose, which appears to have been co-purified with the protein. A comparison between the apo and ligand-bound forms of the same conformational state would be highly informative, as it would allow structural changes in the binding site to be monitored in the absence versus presence of the ligand.

- We apologise for the misunderstanding. We do not have an apo bound form of the cytoplasmic open state of CaVrg4. We are now working on ways to strip the GDP-mannose from the transporter (as assessed using native MS) and we will follow up on this suggestion, very likely combined with the in-depth molecular dynamics suggestion made above.

In the Methods section, the authors note that they improved the ligand density through 3D classification, which suggests that the "unused" classes might correspond to an apo form —

depending on the effectiveness of the classification. If feasible, it would be valuable for the authors to attempt a reconstruction of the apo form from their existing data and to compare it with the ligand-bound structure.

Specific comments:

- Table 1 is a bit minimalistic; it should include Ramachandran statistics and Map sharpening B factors (Å2)
- Done.
- No cryo-EM volume for any of the determined structures (only PDBs and a surface representation of the PDB) is shown in the main figures. Please add this information as a panel to the figures.
- Done.
- The authors should include a directional FSC diagram in the processing figures as well as cFAR values.
- Done.
- A Ligplot-style diagram highlighting the interactions between the ligand and sidechains of the transporter should be added. This will make it easier for the reader to spot crucial interactions.
- Unfortunately, we couldn't get LigPlot to work on any of our Mac systems following the recent upgrades to Tahoe 26. However, we have included the output from PDBe PISA in Extended Data Tables 1 and 2 for the nanobodies, and we have included additional views of the ligand in Extended Data Fig. 7, which we think adequately address this very valid point.
- In the Methods section, the authors note that they improved the ligand density through 3D classification, which suggests that the "unused" classes might correspond to an apo form — depending on the effectiveness of the classification. If feasible, it would be valuable for the authors to attempt a reconstruction of the apo form from their existing data and to compare it with the ligand-bound structure."
- We agree with the reviewer that this may be possible, but unfortunately, the particles from the unused classes could not be combined to generate a volume at sufficient resolution to understand if they are 'unhappy' ligand bound particles or genuinely 'apo'.

Reviewer #4 (Remarks to the Author):

I co-reviewed this manuscript with one of the reviewers who provided the listed reports. This is part of the Nature Communications initiative to facilitate training in peer review and to provide appropriate recognition for Early Career Researchers who co-review manuscripts.

1. Parker, J.L. & Newstead, S. Gateway to the Golgi: molecular mechanisms of nucleotide sugar transporters. *Current Opinion in Structural Biology* **57**, 127-134 (2019).
2. Parker, J.L., Corey, R.A., Stansfeld, P.J. & Newstead, S. Structural basis for substrate specificity and regulation of nucleotide sugar transporters in the lipid bilayer. *Nat Commun* **10**, 4657 (2019).
3. Parker, J.L. & Newstead, S. Structural basis of nucleotide sugar transport across the Golgi membrane. *Nature* **551**, 521-524 (2017).

Reviewer #1 (Remarks to the Author):

In the revised version, Parker et al. added more detailed illustrations of the interaction of the nanobody with Vrg4 and presented more experiments characterizing the nanobody. Moreover, they argued that IC50 and ELISA are as robust as more sophisticated assays, which is fine. They have also made an intensive effort to provide better support for the modeling of the substrate.

The issue with the substrate seems not only to be the occupancy problem, but also flexibility. I still find the binding pose of the substrate quite weird in terms of its interaction with protein side chains, and I reserved my concerns about the accuracy of the modeling of the substrate. Mutagenesis only pinpoints protein residues important for substrate binding, but doesn't provide information on the binding pose of the substrate. It would be helpful for potential readers to understand the current model better by showing the cryo-EM density of the interacting residues, along with the substrate density (it should be a routine presentation). And MD simulation might be useful to test the stability of the current model.

If everyone else thinks the current substrate-binding pose is fine, go ahead with publication.

Our response: We appreciate the referees' comments on this point. Indeed, we have presented the modelled structure of GDP-mannose with careful language to highlight the challenges in modelling this ligand. Specifically, we state 'The maps obtained indicate the binding pose for GDP-mannose is highly flexible, with alternate positions for the sugar ring. Nevertheless, our analysis below is made based on the most likely position of the ligand in the binding site.' and 'Therefore, despite the density being suboptimal for precise ligand modelling, the transport assay results support the proposed location of GDP-mannose in the cytoplasmic-facing state'. We have included the cryo-EM density for the interacting side chains in Supplementary Information Fig. 8b. In a new Supplementary Information Fig. 8c we have shown the B-factor sharpened maps which reveal a break in the ligand density around the beta-phosphate under high threshold values, suggesting increased flexibility of the sugar ring. To further illustrate this point, we have included an alternate pose for the sugar ring in this figure.

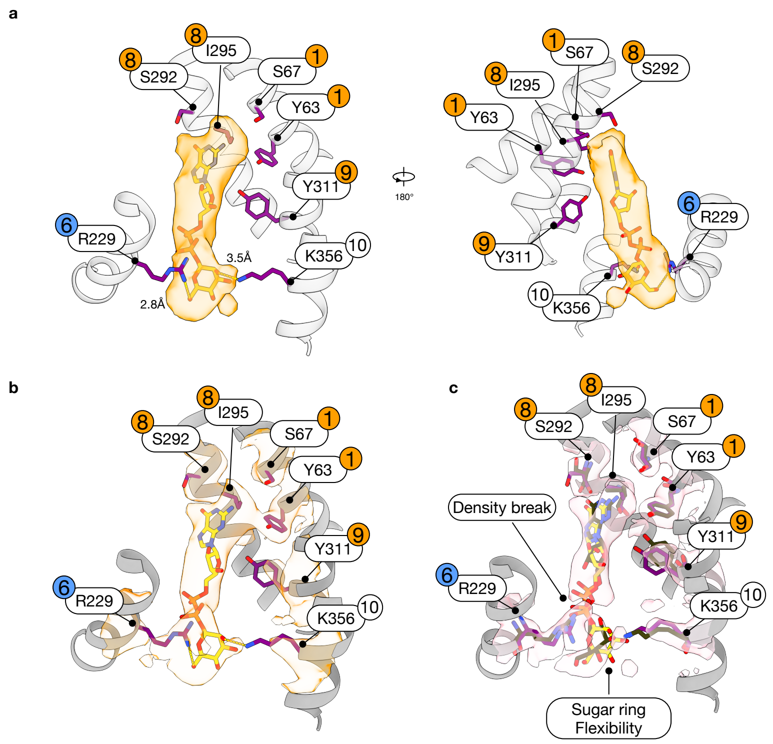

Supplementary Fig 8. Recognition of GDP-mannose in the cytoplasmic-facing state. a, Cryo-EM density of the GDP-mannose ligand (orange, unsharpened map, contoured at a threshold of 0.211). Side chains sitting close (within ~ 4 Å) to the ligand are shown (purple sticks) and labelled with their respective TM helices. Hydrogen bonds to Arg229 and Lys356 are shown and distances labelled. **b,** Cryo-EM density of the side chains modelled interacting with GDP-mannose (orange, unsharpened map, contoured at a threshold of 0.211). **c,** Cryo-EM density following B-factor sharpening (purple, contoured at a threshold of 0.461). Overlaid onto the deposited model is the alternate position of the GDP-mannose ligand (green sticks). Of note, the density in the sharpened maps is broken around the beta-phosphate, indicating increased flexibility of the sugar ring. It is possible the binding of the nanobody restricts the ability of the transporter to fully engage the substrate.

We acknowledge that the use of MD to test this pose is also a useful suggestion; however, as we highlighted in our previous response to this comment, using all-atom MD with transporters is challenging and is likely a separate study in its own right.

Reviewer #2 (Remarks to the Author):

The authors have sufficiently responded to all of my comments. I support the manuscript for publication.

Reviewer #3 (Remarks to the Author):

The authors have satisfactorily answered my questions and considered different suggestions. I recommend the paper for publication.

Reviewer #4 (Remarks to the Author):

I co-reviewed this manuscript with one of the reviewers who provided the listed reports. This is part of the Nature Communications initiative to facilitate training in peer review and to provide appropriate recognition for Early Career Researchers who co-review manuscripts.
